# TcTASV: A Novel Protein Family in *Trypanosoma cruzi* Identified from a Subtractive Trypomastigote cDNA Library

**DOI:** 10.1371/journal.pntd.0000841

**Published:** 2010-10-05

**Authors:** Elizabeth A. García, María Ziliani, Fernán Agüero, Guillermo Bernabó, Daniel O. Sánchez, Valeria Tekiel

**Affiliations:** Instituto de Investigaciones Biotecnológicas (IIB-Intech), Universidad Nacional de General San Martín – CONICET, Buenos Aires, Argentina; New York University School of Medicine, United States of America

## Abstract

**Background:**

The identification and characterization of antigens expressed in *Trypanosoma cruzi* stages that parasitize mammals are essential steps for the development of new vaccines and diagnostics. Genes that are preferentially expressed in trypomastigotes may be involved in key processes that define the biology of trypomastigotes, like cell invasion and immune system evasion.

**Methodology/Principal Findings:**

With the initial aim of identifying trypomastigote-specific expressed tags, we constructed and sequenced an epimastigote-subtracted trypomastigote cDNA library (library TcT-E). More than 45% of the sequenced clones of the library could not be mapped to previously annotated mRNAs or proteins. We validated the presence of these transcripts by reverse northern blot and northern blot experiments, therefore providing novel information about the mRNA expression of these genes in trypomastigotes. A 280-bp consensus element (TcT-E element, TcT-E*elem*) located at the 3′ untranslated region (3′ UTR) of many different open reading frames (ORFs) was identified after clustering the TcT-E dataset. Using an RT-PCR approach, we were able to amplify different mature mRNAs containing the same TcT-E*elem* in the 3′ UTR. The proteins encoded by these ORFs are members of a novel surface protein family in *T. cruzi*, (which we named TcTASV for *T. cruzi* Trypomastigote, Alanine, Serine and Valine rich proteins). All members of the TcTASV family have conserved coding amino- and carboxy-termini, and a central variable core that allows partitioning of TcTASV proteins into three subfamilies. Analysis of the *T. cruzi* genome database resulted in the identification of 38 genes/ORFs for the whole TcTASV family in the reference CL-Brener strain (lineage II). Because this protein family was not found in other trypanosomatids, we also looked for the presence of TcTASV genes in other evolutionary lineages of *T. cruzi*, sequencing 48 and 28 TcTASVs members from the RA (lineage II) and Dm28 (lineage I) *T. cruzi* strains respectively. Detailed phylogenetic analyses of TcTASV gene products show that this gene family is different from previously characterized mucin (TcMUCII), mucin-like, and MASP protein families.

**Conclusions/Significance:**

We identified TcTASV, a new gene family of surface proteins in *T. cruzi*.

## Author Summary

Chagas' disease, caused by the kinetoplastid protozoan parasite *Trypanosoma cruzi*, is endemic in Latin America. At present there are neither vaccines for prevention nor totally effective drugs for the treatment of the disease. *T. cruzi* has a complex life cycle alternating between a reduviid insect (the vector) and a mammalian host, where different parasite stages are found. Differentially expressed genes are the hallmark of the specialized biology of each life cycle stage. The aim of this work was to identify genes expressed in the trypomastigote stage (a blood-circulating stage that invades new cells and spreads the infection in different organs of the mammalian host) that could be used to develop new vaccines or diagnostics. An initial screening of trypomastigote transcripts was performed by sequencing of an epimastigote-subtracted trypomastigote cDNA library. Besides identifying a large proportion of differentially expressed mRNAs, we discovered a novel protein family, which we denominated TcTASV.

## Introduction

*Trypanosoma cruzi*, a kinetoplastid protozoan parasite, is the causative agent of the American trypanosomiasis, also known as Chagas' disease, a zoonotic disease that affects about 8 million individuals in Latin America [1]. The disease is a chronic illness, which symptoms appear 10 or more years after the beginning of the infection, being the most common clinical forms the digestive megas and heart failure, which can lead to death. Currently, there is no effective therapy nor vaccine for the treatment or prevention of the disease [1], [2]. The identification and characterization of proteins expressed in the mammalian stages of *T. cruzi* (amastigotes and trypomastigotes) are key to drug and vaccine development [3].

The genome of the CL-Brener clone of *T. cruzi* was already sequenced by 2005 [4], but its final assembly has only been partially completed recently, mainly because of the high number of repetitive sequences [5]. Although 90% of the genes were assembled in 41 chromosomes, the remaining 10%, the majority of which belong to multigene families, are still excluded from the assembly, as they have not been assigned to any chromosome. Moreover, 64% of the predicted genes have been annotated as hypothetical proteins –their function and/or expression is unknown-, and it is possible that other genes may not have been annotated as genes at all. Therefore, the generation of expressed sequence tags (ESTs, single pass reads obtained from randomly selected cDNA clones) is still a valuable approach to map the location of genes, to obtain experimental evidence about their expression, to identify stage-specific transcripts, and to identify their untranslated regions (UTRs). Previously, we reported the sequencing and analysis of two full-length cDNA libraries constructed from trypomastigotes and amastigotes [6]. Because those libraries were not normalized and were prepared under similar conditions, we were able to identify a number of EST clusters that showed a significantly biased composition in the number of sequences derived from either the trypomastigote and/or the amastigote cDNA libraries. However, only one cluster corresponded to a case of apparent increased expression in trypomastigotes.

In the present work, we focused our attention on the identification of mRNA transcripts over-represented in the mammalian trypomastigote stage as compared to the vector-associated epimastigote stage, using a subtractive PCR approach [7]. Molecules that are differentially expressed in the trypomastigote stage may be involved in the extracellular survival, dissemination to different organs and cell invasion that are the hallmarks of of this parasite stage. Besides finding a large proportion of novel and differentially expressed mRNAs in trypomastigotes (most of them with an unknown function), we discovered a novel protein family, which we denominated TcTASV. The expression profile and the genetic mapping of TcTASVs in the CL-Brener, Dm28 and RA *T. cruzi* strains were also investigated in this work.

## Methods

### Ethics Statement

All procedures requiring animals were performed in agreement with the guidelines of the Animal Ethics Comitee of our Institution.

### Parasites

The CL-Brener clone (reference strain), RA (lineage II) and Dm28 (lineage I) strains of *T. cru*zi were used [8], [9], [10]. Trypomastigotes and amastigotes were obtained *in vitro* by infection of Vero cells grown in Minimum Essential Medium (MEM)-3% foetal bovine serum. For the library construction essentially pure CL-Brener trypomastigotes (with less than 3% amastigote forms) were used. Epimastigotes were obtained from axenic cultures, as previously described [11].

### Construction, sequencing and analysis of the subtractive library TcT-E

Total RNA was isolated from trypomastigotes and epimastigotes with TRIzol (Gibco-BRL) and mRNA purified with polyA-Tract mRNA isolation system (Promega). The PCR-Select cDNA Subtraction kit was used for library construction following the selective subtractive hybridization protocol provided by the manufacturers (CLONTECH). First strand cDNA synthesis was performed with 2 µg of polyA+ of each *T. cruzi* stage (trypomastigote and epimastigote), oligo dT primer with a 5′ RsaI site and Superscript II reverse transcriptase (Gibco-BRL). Second strand cDNA synthesis was performed with T4 DNA polymerase. After RsaI digestion of double stranded cDNA, two different sets of adaptors were ligated to the tester cDNA (trypomastigotes) but not to the driver cDNA (epimastigotes). Two rounds of subtractive hybridization in the presence of an excess of driver cDNA were performed, thus leading to the enrichment of differentially expressed sequences in the tester cDNA population that were the templates for further suppressive PCR amplification performed with adaptor-specific primers [7]. The subtraction efficiency was verified by monitoring the PCR amplification of the *T. cruzi* histone 2A transcript in subtracted and unsubtracted samples (H2_3′: tcttggacgccttcttcgct; H2_5′: gtgatgccgagcctgaacaa). PCR products enriched for tester differentially expressed sequences -higher than 100 bp- were cloned into the pGEM T-Easy vector (Promega). *E. coli* DH5α cells were transformed with ligations; white colonies were randomly picked and the TcT-E library plated in 384-well microplates.

Template preparation of clones for sequencing was carried out as previously described [12]. Sequencing reactions were performed in a Perkin Elmer 9600 thermal cycler by using a Dye Terminator Cycle sequencing Ready Reaction Kit with AmpliTaq DNA polymerase according to the protocols supplied by the manufacturer (Applied Biosystems). Single-pass sequencing was performed on an ABI 377 automated sequencer.

Bases were called by PHRED and an automated protocol for the analysis of the data was used to assess sequence quality and trim vector, adaptors and unreliable data from sequences [6]. Sequences longer than 100 bases were further analyzed. Sequence similarity searches against in-house databases were run locally using the BLAST suite of programs as distributed by the NCBI in a PC computer running Linux. Sequences were also compared against the NCBI non-redundant protein or nucleotide databases by using BLASTX or BLASTN programs respectively (cut off values: BLASTN p<10e-40; BLASTX p<10e-5) [12], [13].

### Northern, reverse northern blot and southern blot analysis

For Northern blot, total RNA (20 µg/lane) from trypomastigotes and epimastigotes was electrophoresed on 1.5% agarose formaldehyde gels and transferred to nylon membranes (Zeta-Probe, BioRad). All TcT-E clones used as probes were labeled with ^32^P by PCR using adaptor-specific primers (Nest_2R: agcgtggtcgcggccgaggt; Nest_1: tcgagcggccgcccgggcaggt). Hybridization and washing were performed at 63°C following standard procedures [14]. The complete ORF *Tcruzi_1863-4-1211-93* (TriTrypDB.org) was amplified by PCR from the clone G53E20 (GenBank Acc AZ050960) from a random genomic library DNA [12], labeled by PCR and used as probe in northern and southern blot experiments.

For reverse northern blots, clones of the TcT-E library were picked, grown in LB-ampicillin in 96-well plates and subjected to colony-PCR using 1 µl of culture and primers Nest_2R and Nest_1 [15]. The sizes of the inserts were checked on a 2% agarose gel and PCR products were then denatured and dotted in duplicate onto nitrocellulose membranes. Filters were hybridized with cDNA probes synthesized from total RNA of trypomastigotes and epimastigotes by reverse transcription using ^32^P-dCTP. Plasmids containing tubulin and SAPA (shed acute phase antigen) *T. cruzi* genes were dotted on membranes as positive controls, whereas a plasmid containing a non-related (mouse) gene was used as a negative control.

For southern blots, DNA was prepared from epimastigotes of the CL-Brener strain by using the conventional Proteinase K phenol-chloroform method and digested with the indicated restriction enzymes. Electrophoresis, hybridization and washing were performed by standard procedures [14].

### Experimental identification of TcTASVs and TcT-E element

The complete TcT-E element (TcT-E*elem*) was obtained from CL-Brener genomic DNA by PCR using Pfu DNA polymerase and the primers TcT-Ee_pp_Hind (taaagcttccgggcaggtacagtat) and TcT-Ee_pp_Xho (atctcgagtgagaatcccgcaggact).

Mature mRNA transcripts containing both the TcT-E*elem* and the different upstream open reading frames (ORFs) were identified by RT-PCR and sequencing in the CL-Brener strain. RNA was treated with RQ1 DNase (Promega Corp., Madison, USA) and first strand cDNA synthesized using an oligo dT primer. PCR was performed using a 5′ primer specific for the *T. cruzi* miniexon containing an EcoRI site (cccgaattcaacgctattattgatacagtttctgt) and a 3′ antisense primer corresponding to the 3′ region of the TcT-E*elem* (TcT-Ee_int_R: aagaaatgattcggcaggaa). PCR products were gel- excised, purified using QIAex II (Qiagen) and cloned. Alternatively, after first strand cDNA synthesis, PCR was performed with primers corresponding to the 5′ and 3′ conserved regions of the majority of the ORFs (CDS_desc_L: gtcgagcgactctacgacg; CDS_desc_R: acagcagcacagacaaggg) or with the 5′ CDS_desc_L and the 3′ T-Ee_int_R primers. Bands were also gel-excised, cloned and sequenced. Conceptually translated proteins corresponding to the cloned CDS were aligned by the Clustal method.

To search for TcTASV in other *T. cruzi* strains, genomic DNA from Dm28 (lineage I, currently *T. cruzi* I) and RA (lineage II, currently *T. cruzi* VI) was amplified by PCR using primers CDS_desc_L and CDS_desc_R [8], [9], [10]. The bands obtained were gel-purified, cloned and sequenced on both strands on an ABI 3130. The sequences of each clone were assembled using the program DNAbaser. Phylogenetic trees were constructed from amino acid alignments using the Neighbour Joining method, and bootstrapped using 1000 permutations. The trees were rooted using 6 sequences as outgroups, and were visualized with the TreeView program (http://taxonomy.zoology.gla.ac.uk/rod/treeview.html).

### *In silico* analysis of TcTASV and TcT-E*elem* sequences

The nucleotide sequence AF080220 (GenBank Accession number) was used to carry out a BLASTN search against the TcT-E database. A multiple sequence alignment was computed using the Clustal Method [16]. The consensus sequence of the TcT-E element (Tc*T-Eelem*) was used as bait to search the unassembled whole genome shotgun sequences (GSSs) of *T. cruzi* at TIGR (http://tigrblast.tigr.org/er-blast/index.cgi?project=tca1). GSSs identified in this way were assembled into contigs, that were then visualized and edited in Artemis to identify *in silico* additional TcTASVs [17]. Motif scanning for signal peptide, cleavage sites (SignalP) and Ser, Thr, and Tyr phosphorylation sites (NetPhos) was performed in the ExPASy proteomics server at http://www.expasy.org/. The prediction of glycosylphosphatidylinositol (GPI) anchor addition sites, was performed using DGPI (run locally) and FragAnchor (http://navet.ics.hawaii.edu/~fraganchor/NNHMM/NNHMM.html) [18].

### TcTASV-A peptide and generation of antiserum

The peptide RQ28 (GKLRWRFQGEKDWRKC) comprising amino acids 57 to 72 of TcTASV-A1 (GenBank AM492199) was purchased from Sigma-Genosys. This sequence was chosen because it is present in the conserved, noncleaved N-terminal region of the protein family which is also predicted not to be glycosylated or modified. The KLH coupled peptide was used to develop an anti-TcTASV-A specific serum in rabbit. Total IgG from anti-RQ serum was purified with protein G columns (HiTrap, GE Healthcare Life Sciences) and specific anti-TcTASV-A antibodies were purified by column affinity with SulfoLink Kits coupled with the RQ28 peptide (Thermo Scientific). Antibodies were used at 0.1 µg/ml.

### TcTASV expression in *T. cruzi*

Protein extracts of *T. cruzi* epimastigotes, trypomastigotes and amastigotes were resuspended in cracking buffer (60 mM Tris-HCl pH 6.8; 2% SDS, 0.1% glycerol, 5% â-mercaptoethanol) in the presence of protease inhibitors at a density of 1–2×10^6^ parasites/µl. Conventional SDS-PAGE was performed on 12% polyacrylamide gels, and proteins were then transferred to nitrocellulose filters. Blots were incubated with anti-TcTASV-A antibodies, washed, incubated with an anti-rabbit secondary antibody labelled with horseradish-peroxidase (DAKO) and developed with chemiluminescence.

**Note:** Nucleotide sequence data reported in this paper have been submitted to the EMBL/GenBank/DDBJ databases with the accession numbers AM492199–AM492211, GW883555–GW883875, HO052091–HO052172 and FN599093–FN599167.

## Results

### Most of the clones in the epimastigote-subtracted trypomastigote cDNA library (library TcT-E) are new expression tags that are specific for the trypomastigote stage of *T. cruzi*

**Figure 1:**
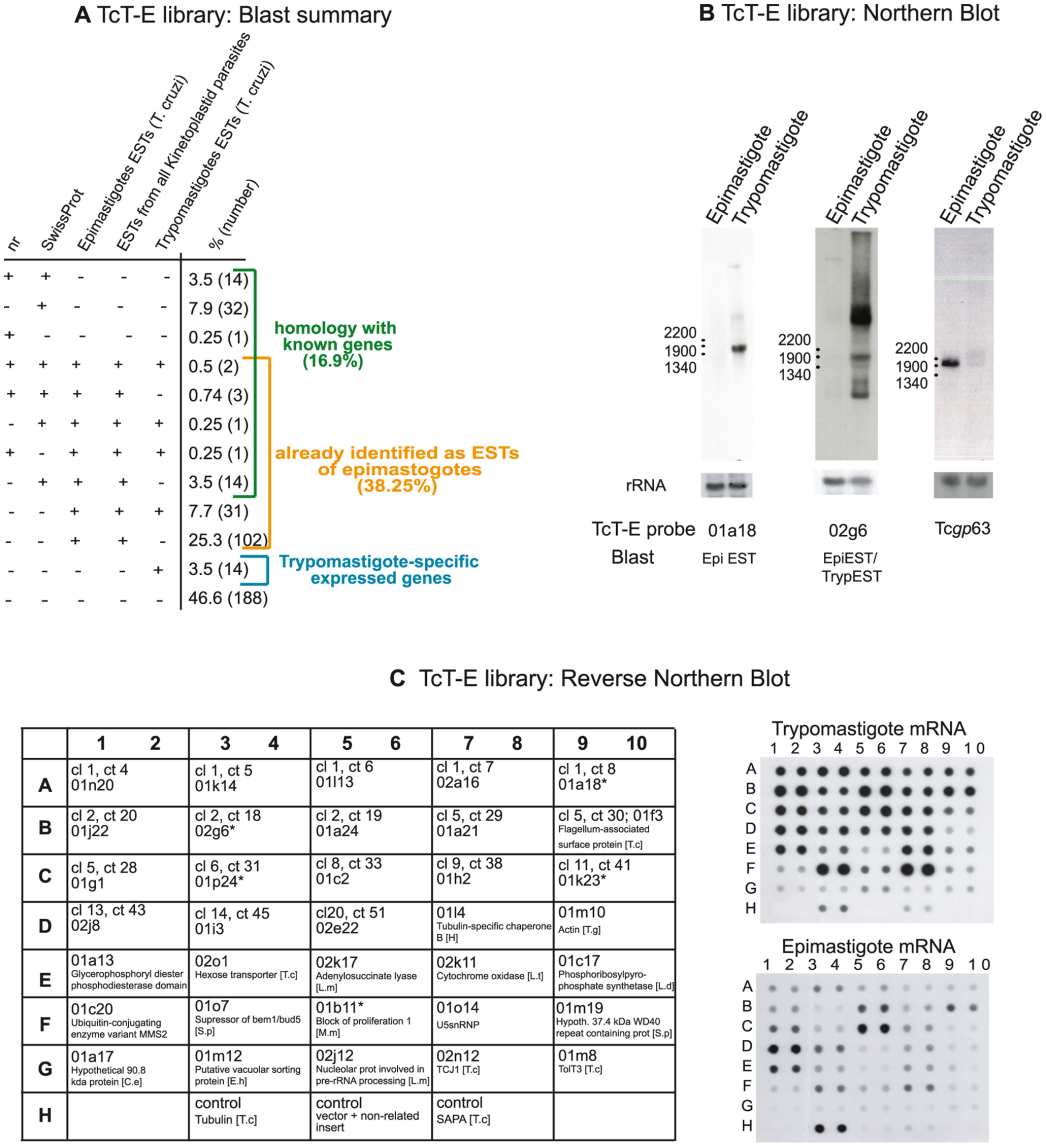
TcT-E: *Trypanosoma cruzi*, epimastigote-subtracted trypomastigote cDNA library; overview. **(A). Sequence similarity analysis of TcT-E clones against protein and ESTs databases.** All 403 sequenced TcT-E ESTs were compared to sequences in protein databases (nr, SwissProt) by BLASTX and to kinetoplastid ESTs by BLASTN. Matches were considered significant if they showed an E value<10^−5^ for proteins and <10^−40^ for DNA. A table listing the detailed matches is provided online at http://genoma.unsam.edu.ar/projects/tct-e/tct-e.p.html. **mRNA corresponding to TcT-E clones is preferentially expressed in trypomastigotes.** **(B) Northern blot:** Total RNA (20 µg/lane) from *T. cruzi* trypomastigotes and epimastigotes was electrophoresed and blotted on nylon membranes by standard procedures. Each blot was hybridized with one of the indicated TcT-E clones as probe. Tcgp63-I was used as control of epimastigote preferential mRNA expression [55]. To check equal loading of RNA, the membranes were stripped and rehybridized with a *T. cruzi* 24S ribosomal probe (bottom panel). **(C) Reverse northern blot:** Thirty-five TcT-E clones were picked, amplified by PCR and dotted on nylon membranes in duplicate. Two identical membranes were prepared and hybridized with trypomastigote and epimastigote ^32^P-labelled first-strand cDNA (exposure time: 24 h). The scheme on the left indicates which TcT-E clone (i.e. 01n20, 01k14, etc) was dotted in each position of the membrane as well as the corresponding cluster and contig (i.e. cl1, ct4; cl1, ct5; etc). When appropriate, the BLAST result of the TcT-E clone is also indicated. Controls were dotted on row H: columns 3 and 4: TcTubulin; columns 5 and 6: pGEM-T Easy with non-related insert; columns 7 and 8: TcSAPA. Abbreviations: C. elegans: C.e.; E. histolytica: E.h.; T. gondii: T.g.; T. cruzi: T. c.; S. pombe: S.p.; L. major: L.m.; L. tarentolae: L.t.; L. donovani: L.d; Human: H; M. musculus: M.m.

To gain information about the genes that are differentially expressed in the trypomastigote (circulating stage in mammals) but not in the epimastigote (replicative stage in the insect vector) of *T. cruzi*, we built a library of trypomastigote cDNA subtracted with epimastigote cDNA (TcT-E library). Partial sequencing of this library provided high-quality sequences of 403 clones (GenBank Acc GW883555–GW883875 and HO052091–HO052172). With this set of data sequence (BLAST) analyses were performed against various databases of trypanosomatid ESTs (*T. cruzi* trypomastigotes, *T. cruzi* epimastigotes, ESTs of all kinetoplastids) and against protein databases (nr at GenBank and SwissProt) (Fig. 1A). The BLAST reports for all searches can be accessed online at http://genoma.unsam.edu.ar/projects/tct-e/tct-e.p.html (Table S1). Briefly, more than 46% of the TcT-E dataset do not have any known mRNA or protein homologue and only two clones give positive hits against all databases (Fig. 1A).

The comparison of the TcT-E dataset against *T. cruzi* ESTs showed that (a) only 3.5% of them corresponded to trypomastigote-specific tags that were identified prior to this work and (b) 38% of TcT-E clones have significant matches with epimastigote sequences (Fig. 1A). The latter was expected in part because of the high number of epimastigote ESTs available in public databases. This could also indicate that these transcripts, although present in both stages, might be expressed at a higher level in trypomastigotes than in epimastigotes, since our library had been subtracted with epimastigote cDNA. By Northern blot (Fig. 1B) and reverse Northern blot on 86 randomly picked TcT-E clones (data not shown), we were able to confirm that even in those cases were a TcT-E clone had identity with ESTs obtained from epimastigotes, the mRNA levels were consistently higher in trypomastigotes than in epimastigotes. Sixty-eight (16.9%) clones had similarity to known proteins (nr and SwissProt databases) and 33 of them matched previously described trypomastigote antigens like the flagellum-associated surface protein FL-160 (gb|AAA30196) or sialidase homologues (AF051695 and AF051696) (Table S1). The top 50 hits against SwissProt and GenBank (nr) are provided in Table S2. The whole TcT-E dataset can be searched by blast at http://genoma.unsam.edu.ar/projects/tct-e/.

To compensate for sequencing errors and to obtain longer sequences, we next generated a non-redundant TcT-E EST set by clustering (using the *blastclust* tool from the NCBI C Toolkit), which was composed of 23 clusters containing 261 sequences and 142 singletons (ESTs with no similarity against any other EST) (Table S3). EST clones belonging to clusters with the largest number of sequences as well as other clones that showed significant similarity to SwissProt and/or GenBank nr databases were selected to analyze their expression by reverse northern blot (Fig. 1C). We observed that most of the TcT-E clones were actually overexpressed in trypomastigotes, which again confirms the correct subtraction of the library, and that the sequences generated provide information about the transcripts differentially expressed in the trypomastigote stage.

### A 280-bp element that is highly represented in the TcT-E library is found at the 3′ UTR of several genes

**Figure 2:**
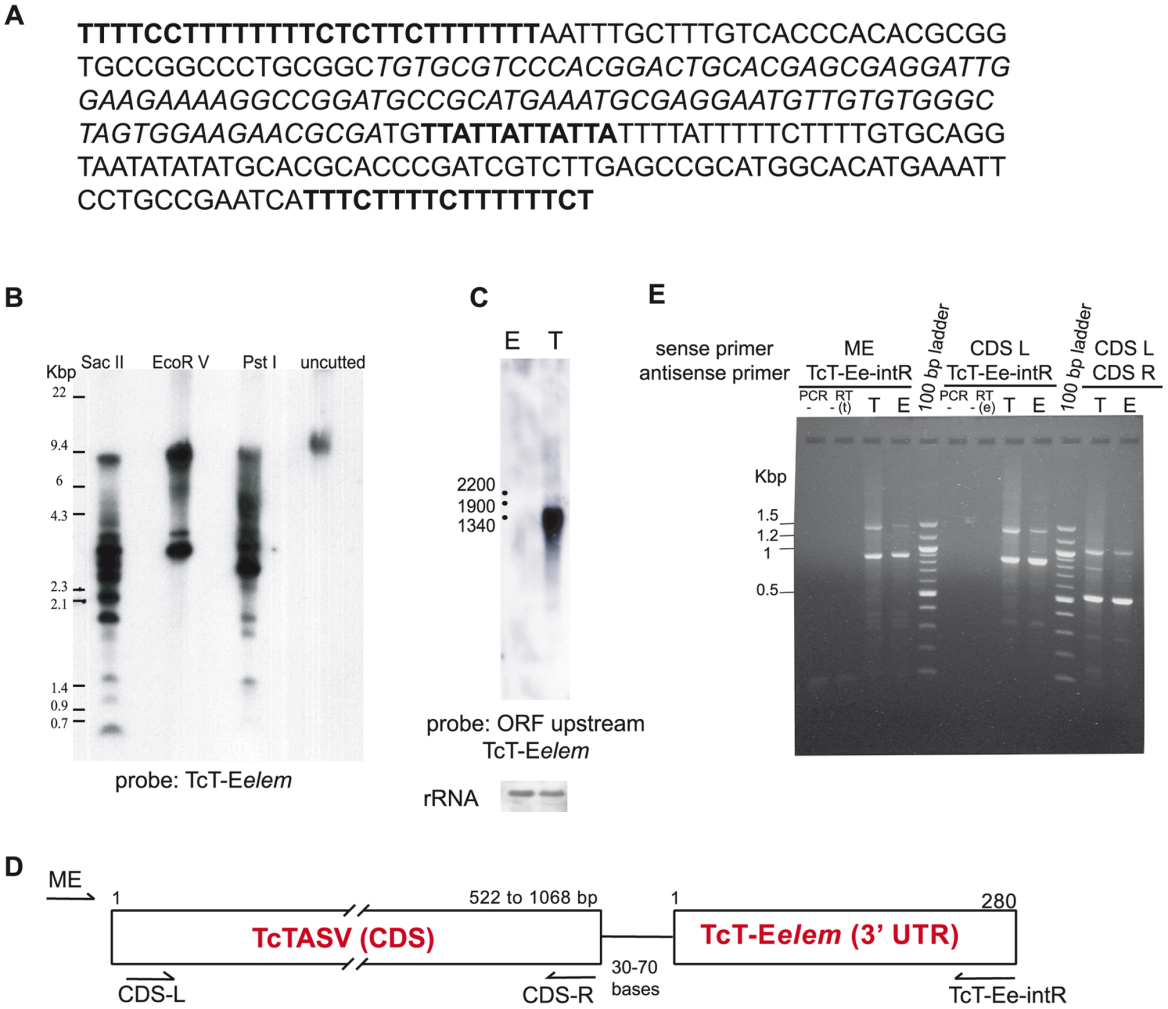
The TcT-E element (TcT-E*elem*) is present in multiple copies in the *T. cruzi* genome and is associated with different coding regions. **(A) Identification of an enriched 280-bp element in the TcT-E library.** *In silico* screening of the TcT-E library using the FL-160-2 3′ UTR as bait depicted a large number of clones displaying homology with nucleotides 372–472. After analyzing a multiple sequence alignment of the identified TcT-E clones, a 280-bp consensus sequence with 3′ and 5′ polypyrimidine tracts (bold) and a variable number of TAA repeats (bold underlined) was obtained, and defined as TcT-Eelement (TcT-E*elem*). **(B) Analysis of TcT-E** ***elem*** **copy number.** *T. cruzi* genomic DNA (CL-Brener strain) was digested with restriction enzymes having no internal site within the TcT-E*elem*, electrophoresed on TAE-agarose gel and transferred by standard procedures. A probe specific for the TcT-E*elem* was synthesized and labelled by PCR with ^32^P. **(C) The mRNA of the CDSs located upstream of the TcT-E are preferentially expressed in trypomastigotes.** Northern blots probed with the complete ORF *Tcruzi_1863-4-1211-93* (http://TriTrypDB.org). **(D) The TcT-E** ***elem*** **is present 30–70 bp downstream of a stop codon in many coding sequences in** ***T.cruzi*** **.** The consensus sequence of TcT-E*elem* was used as bait to search the *T. cruzi* database; WGSs with more than 80% identity to TcT-E*elem* and longer than 1000 bp were retrieved and analyzed. The schematic map of the relative position of TcT-E*elem* in relation to coding sequences in *T. cruzi* genome is shown. **(E) Mature mRNA transcripts contain both the TcT-E** ***elem*** **and different CDSs.** Total RNA from trypomastigotes (T) and epimastigotes (E) was purified and treated with RQ1 DNase. First strand cDNA was synthesized by RT using an oligo dT primer. PCR was performed using a 5′ primer specific for the *T. cruzi* miniexon (ME) and a 3′ antisense primer corresponding to the 3′ region of the TcT-E*elem*. Alternatively, PCR was performed with primers corresponding to the 5′ and 3′ conserved regions of most CDSs (CDS-L and CDS-R) or with a 5′primer specific for the CDS and a 3′ primer specific for the TcT-E*elem*. The relative position of the primers is indicated in Fig. 2D. PCR- and RT- denote the negative controls for each reaction.

The larger groups of sequences in the clustered TcT-E EST dataset contained sequences that had no similarity against sequences in other databases. To further characterize these sequences, we then attempted to find any motif or conserved sequence in these contigs. By lowering the cut off value for BLASTN (e≤10e-5), we found that some contigs in cluster 1 showed similarity with a 100-bp region in the 3′ untranslated region (UTR) of the flagellar *T. cruzi* FL-160-2 gene (GenBank Acc AF080220) [19], [20]. By computing a multiple sequence alignment of the TcT-E clones that presented identity in these 100 bases, we reconstructed a consensus sequence of 280 bp (Fig. 2A). We named this 280-bp element ***TcT-E element*** (TcT-E*elem*) because of its high representation in the subtractive TcT-E library. The first 27 and last 17 bases of the TcT-E*elem* (bold in Fig. 2A) are polypyrimidine tracts and bases 66 to 165 (in italic) correspond to those similar to the 3′ UTR of FL-160-2. Another feature of the TcT-E*elem* is the presence of a variable number (between 3 and 5) of TTA repeats (bold underlined in Fig. 2A). By southern blot, we found a pattern indicative of multiple genomic copies of the TcT-E*elem* since several bands were detected, even though none of the restriction enzymes used are predicted to cut into the probe (Fig. 2B). Because the similarity of the TcT-E*elem* to the 3′ UTR of the FL-160 gene was limited to 100 bp out of the 280 bp of the TcT-E*elem*, we reasoned that TcT-E*elem* might be associated to other genes (i.e. be present in gene contexts other than FL-160 genes).

The sequence of the TcT-E*elem* was used to search the *T. cruzi* genome raw data (unassembled whole genome shotgun sequences, or contigs assembled by the genome project) and the position of the TcT-E*elem* relative to upstream open reading frames (ORFs) was determined. Interestingly, the polypyrimidine tracts contained within the TcT-E*elem* were always found 30–70 bases downstream of the stop codon of a coding region (CDS), in different contigs. The close proximity of the TcT-E*elem* to the end of the upstream coding sequence strongly suggested that the TcT-E*elem* was part of the 3′ UTR of the gene. By southern blot, using a probe corresponding to the complete ORF of a predicted protein associated with the TcT-E*elem* (currently identified as ORF *Tcruzi_1863-4-1211-93*, TriTrypDB database [21]), we observed a hybridization pattern similar to that obtained using the TcT-E*elem* as probe (Fig. 2B), thus reinforcing the genetic linkage between the TcT-E*elem* and the identified ORFs (data not shown). By northern blot analysis we confirmed that, like most of the ESTs from the TcT-E library, this ORF was also differentially expressed in the trypomastigote stage (Fig. 2C). Interestingly, fragments of several of the CDSs found associated with the TcT-E*elem* in this bioinformatic analysis were also represented in the TcT-E library (for example clones TcT-E01p24 and TcT-E01k23, corresponding to GenBank GW883736 and HO052122, respectively, Fig. 1C).

### The new TcTASV protein family has the TcT-E element as part of its 3′ UTR

**Figure 3:**
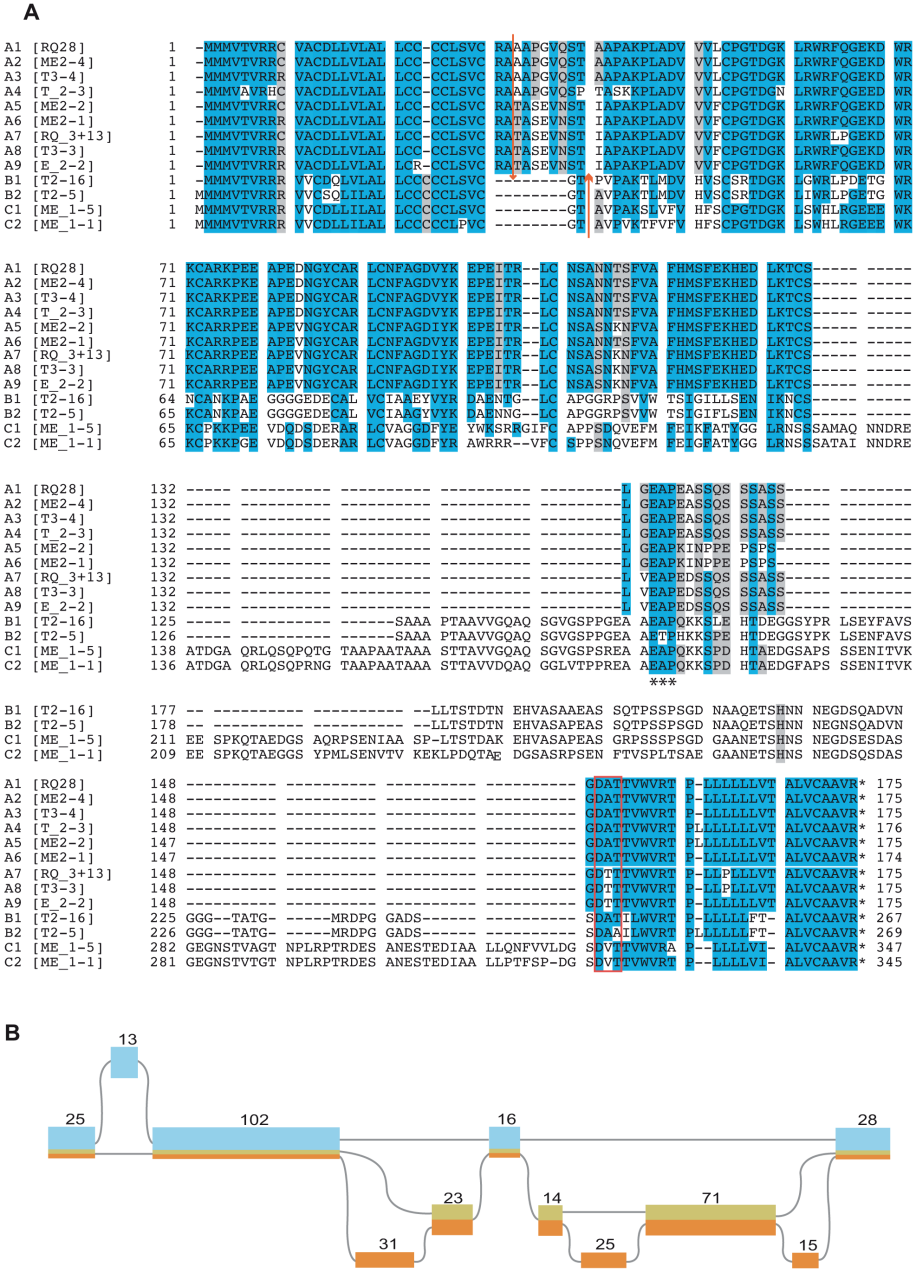
The coding regions associated with the TcT-E*elem* constitute the novel TcTASV protein family that could be grouped into three sub-families. (**A**) PCR products of Fig. 2E were cloned and sequenced. The 13 different mRNA sequences obtained were conceptually translated and aligned by the Clustal method. A broad protein family with all members sharing conserved N- and C- termini, a signal peptide (arrow shows the predicted cleavage site) and a consensus sequence for the addition of a GPI (red box) is made up by all identified proteins. Three sub-families (A, B, C) were defined according to sequence identity and the predicted relative molecular mass (Mr) of the proteins. RQ and ME denote clones derived from a PCR made with ME/TcT-E*elem* primers; T or E denote that the PCR was performed with CDS-L/CDS-R primers using trypomastigote or epimastigote cDNA as template, respectively. The complete mRNA sequences were deposited at GenBank under the accession numbers AM492199–AM49211. (**B**) Overall structure of TcTASV genes. The positions where the sub-families match and diverge in the alignment are presented according to blocks of deletions/insertions of eight amino acids as predicted by the Partial Order Alignment algorithm (and visualized using POAVIZ [25]). The length of the segments and numbers above them indicate the number of amino acids residues contained in each block. A unique color is assigned to each sequence (or to closely-related sequences) in the alignment and the height of the rectangle corresponds to the number of sequences grouped under the same colour (TcTASV-A: ligth blue; TcTASV-B: green; TcTASV-C: orange).

A schematic diagram of the CDS - TcT-E*elem* arrangement found by *in silico* analysis is shown in Figure 2D. Although different coding sequences were located upstream of the TcT-E*elem*, we observed that the amino- and carboxy–termini of those conceptually translated proteins were conserved, suggesting that these ORFs are members of the same family. Besides, we detected three bands by northern blot when using the clone TcT-E01k23 (GenBank HO052122) as probe, which corresponds to the last 270 nucleotides of one TcT-E*elem*-associated ORF (not shown). Thus, both *in silico* and experimental observations suggested the presence of a new protein family sharing conserved amino- and carboxy-termini and the 3′ UTR of the mRNA (TcT-E*elem*). To further investigate this hypothesis we looked for the presence of full-length transcripts, by performing RT-PCR experiments using a reverse primer specific for the 3′ end of the TcT-E*elem* (TcT-Ee-intR) and a forward primer specific for the *T. cruzi* miniexon (ME) that is added by *trans*-splicing to all RNA PolII transcripts in *T. cruzi* [22]. In parallel, other RT-PCR reactions were designed to amplify the CDSs codifying for this new protein family irrespectively of their untranslated region, using the primers indicated in Figure 2D. Bands of ∼1500 bp and ∼900 bp obtained with primers ME/ TcT-Ee-intR as well as the three bands obtained in trypomastigotes with CDS-L/CDS-R primers (Fig. 2E) were cut from the gel, cloned and sequenced. Thirteen different transcripts were identified and deposited at GenBank with the accession numbers AM492199–AM492211. The coding region of the transcripts was conceptually translated and aligned, confirming that they belong to a multigene family with conserved amino- and carboxy-termini and a variable central core (Fig. 3A). The proteins are enriched in Ala, Ser and Val residues, and therefore, the family was named TcTASV (for **T**rypomastigote **A**lanine **S**erine **V**aline rich protein). According to the length of the central region, we were able to define three subfamilies (A, B and C, see Fig. 3A), with a conserved Glu-Ala-Pro motif in the variable region (asterisks in Fig. 3A).

A visualization of the alignment in Fig. 3A using the partial order multiple sequence alignment visualizer POAVIZ (Fig. 3B) [23] helps to define the overall structure of how sequences match and diverge in the alignment, and facilitates the identification of complex branching structures, such as domains or large-scale insertions/ deletions. Aligned regions are joined together in the partial order graph whereas regions that are unaligned are separated, clearly showing the shared and divergent regions of the 3 TcTASV subfamilies, schematically represented in light blue (TcTASV-A), green (TcTASV-B) and orange (TcTASV-C) (Fig. 3B).

The predicted molecular weights of the subfamilies are 18 kDa, 27 kDa and 36 kDa for the A, B and C apoproteins respectively. All proteins had a predicted signal peptide (arrows above and below the alignment in Fig. 3A show the predicted cleavage site) and a consensus sequence for the addition of a GPI anchor (red box in alignment), suggesting that the proteins could be located at the parasite surface. A high proportion of Ser and Thr that could be glycosylated (as found for other surface proteins of *T. cruzi*) were also identified [24], [25].

Surprisingly, many TcTASVs genes were not annotated as genes in the *T. cruzi* genome (available at http://TriTrypDB.org) [4], [5], [21]. For example, only the TcTASV-A 2, 4 and 8 genes (GenBank AM492200, AM492209 and AM492210, respectively) were annotated as protein coding genes (hypothetical), while all other TcTASVs-A were found as unannotated ORFs in the data base (Table S4). Besides, most TcTASVs-A have been annotated starting in an ATG codon (Met residue) located ∼145 aa upstream of the one we identified here as the site of *trans*-splicing, based on the amplification of mature mRNAs using a primer specific for the 5′ spliced leader. The best hits found in TriTrypDB for each TcTASV gene and the corresponding additional information (assigned gene number, contig, identity and other observations about the annotation of these genes is presented in Table S4.

### The new TcTASV family of proteins is found only in *T. cruzi* and is conserved among the parasite lineages

TcTASV genes are only present in *T. cruzi*. Sequence similarity searches revealed no orthologues in the genomes of *T. brucei* and *Leishmania spp*, and in ESTs obtained from *T. rangeli*, the most closely-related trypanosomatids. Based on this observation it is possible to hypothesize that TcTASVs may be involved in *T. cruzi*-specific strategies of survival and/or immune evasion.

Although we obtained experimental evidence supporting the presence of 13 TcTASVs in CL-Brener (nine TcTASVs-A, two TcTASVs-B and two TcTASVs-C), it is likely that the TcTASV family is composed by a higher number of members. Recently, Arner *et al.* developed a public database specifically designed for the identification of repeated genes in the *T. cruzi* genome [26], the assumption being that the genes present in high copy numbers were collapsed during the assembly. By using this resource, the estimated number of genes was predicted to be 14 for TcTASVs-A, 6 for TcTASVs-B and 22 for TcTASVs-C. Our own detailed inspection of the *T. cruzi* data base allowed us to identify 20 TcTASVs-A, 5 TcTASVs-B and 13 TcTASVs-C members, giving a total number of 38 genes for the TcTASV family (Table S5, additional material). In the case of TcTASV-A and B families, when predicted as genes, they were annotated as hypothetical proteins. However, 6 out of 7 TcTASV-C genes were annotated as mucin-like genes. The mucin-like family is another family of surface proteins in *T. cruzi* and, as currently annotated in TriTrypDB, is composed of 28 genes [21]. Although the overall structure of mucin-like genes (conserved amino- and carboxy-termini, predictions for signal peptide and GPI anchor addition) resembles the one for TcTASV genes, mucin-like and TcTASV have very different amino acid composition. The hypotheses that (a) TcTASV is a protein family different from the mucin-like gene family, and (b) the genes that we identified here as TcTASV-C (but were annotated as mucin-like) are indeed members of the TcTASV family and not of the mucin-like family, were tested by a phylogenetic analysis. Starting with an alignment that included all TcTASV and mucin-like genes, we computed a neighbor-joining phylogenetic tree (Figure S1). The tree clearly shows two major branches: one for mucin-like genes and another for TcTASVs genes (including these 6 incorrectly annotated mucin-like genes). On the other hand, the monophyletic origin of TcTASVs in relation to other structurally similar protein families (TcMUCII, mucin-like, and MASP), was also tested through a phylogenetic analysis of 15 sequences from each family (Figure S2) [4], [27], [28], [29].

**Figure 4:**
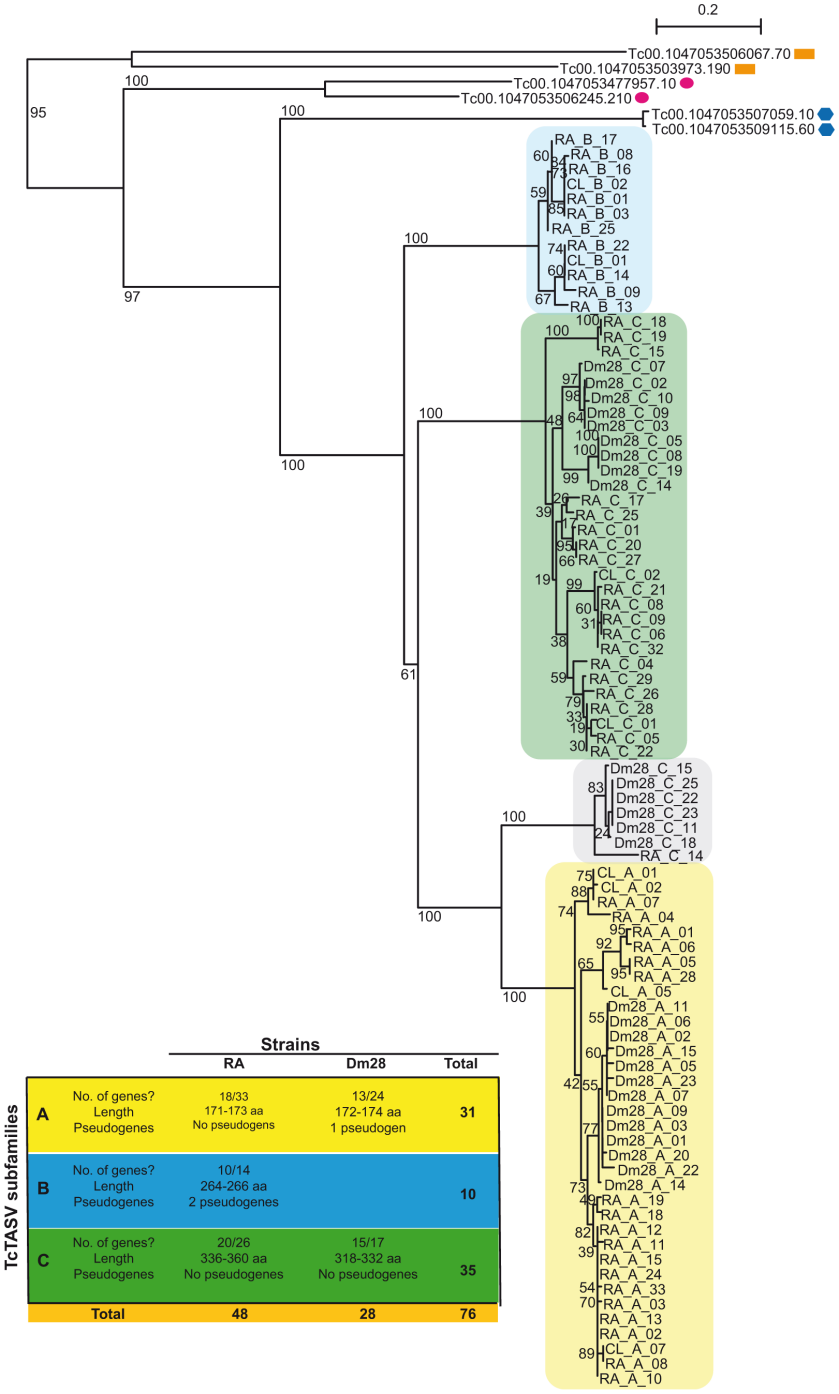
The TcTASV family is conserved among different *Trypanosoma cruzi* lineages. Genomic DNA from Dm28 (lineage I) and RA (lineage II) *T. cruzi* strains was amplified by PCR using primers for the conserved TcTASV 3′ and 5′ regions. The bands obtained were gel-purified, cloned, sequenced on both strands and assembled. **Table**. Summary of TcTASV diversity in the RA and Dm28 strains. The ratios indicate the number of different sequences among the total number of clones analyzed. **Phylogram**. A phylogenetic tree was constructed from amino acid alignments using the Neighbour-Joining method, bootstrapped using 1000 permutations, and rooted using sequences of 3 *T. cruzi* glycoprotein families as outgroups. Sequences are coloured according to the TcTASV subfamily to which they belong. Outgroups: MASP (Tc00.1047053506067.70 and Tc00.1047053503973.190; orange rectangles); TcMUCII (Tc00.1047053477957.10 and Tc00.1047053506245.210; pink circles); mucin-like (Tc00.1047053507059.10 and Tc00.1047053509115.60; blue hexagons).

The limited phylogenetic distribution of the TcTASV family (so far only detected in *T. cruzi*), prompted us to investigate the presence of TcTASV genes in *T. cruzi* strains from other evolutionary lineages (*T. cruzi* I and II). For this, we amplified the genes of the TcTASV family from two representative strains (Dm28 and RA, respectively) using primers specific for the 3′ and 5′ conserved regions. Each of the amplicons obtained for each strain and for each TcTASV subfamily, was cloned to build a mini-library, in order to identify as many members as possible. We obtained 73 clones from the RA strain and 41 from Dm28 strain, but, for further analysis, we selected only those who presented unique sequences for each strain (RA: 48; Dm28: 28; GenBank Acc FN599093–FN599167) (Fig. 4, Table). The 76 unique sequences obtained for RA and Dm28, together with 8 sequences of CL-Brener (TcTASV-A: 4, TcTASV-B: 2 and TcTASV-C: 2) were used to compute a phylogenetic tree, using sequences of other *T. cruzi* glycoprotein families (mucin-like, TcMUCII and MASP) as outgroups (Fig. 4). All three (TcTASV-A, B and C) subfamilies were identified in this dataset. We also identified a new subgroup composed of six Dm28 and one RA sequences with mixed characteristics that could constitute a new TcTASV subfamily with some characteristics shared with members of the A subfamily (amino acid sequence) and others shared with members of the C subfamily (length) (Fig. 4, gray box). As in the case of the CL-Brener strain, the subfamilies with most members were TcTASV-A and TcTASV-C and, interestingly, we noted the absence of the TcTASV-B subfamily in the Dm28 strain, which could be explained either by the absence of TcTASV-B genes in this strain or by the accumulation of mutations that prevented the amplification of members of this subfamily in our PCR experiments. Another distinguishing characteristic between the two evolutionary lineages of *T. cruzi* is that proteins of the subfamily C found in lineage II (RA and CL-Brener) are longer than the same proteins found in lineage I (Dm28) (Fig. 4, Table).

To assess the expression of TcTASV family, we took advantage of proteomic data, available in TcruziDB/TriTrypDB, together with experimental data obtained in this work. Mass spectrometry data strongly suggest the differential expression in trypomastigotes of al least 1 out of 4 TcTASV-A genes -Tc00.1047053506337.80, Tc00.1047053506337.100, Tc00.1047053510717.10 and, Tc00.1047053510717.20- that share a peptide that was detected only in this parasite stage (KPGEYESVTDDCAR, 2 spectra) (Table S5) [28], [30]. On the other hand, proteomic evidence of the expression of the TcTASV-A8 gene (GenBank AM492210; Tc00.1047053506573.5) has been reported for trypomastigotes (five mass spectra) and amastigotes (one spectrum) [28] and can be accessed through TcruziDB.org [30]. The peptide identified is also 100% identical to amino acids 89–104 of other TcTASV gene products (A9: GenBank AM492211, A7: GenBank AM492202 and A5: GenBank AM492201). Moreover, the peptide is completely conserved (15/16 identical aa) in all the other TcTASV-A members.

The expression pattern of members of the TcTASV-A subfamily was also analyzed using affinity-purified antibodies that had been generated against a peptide that is conserved throughout the subfamily (see Methods). We were able to find TcTASV-A proteins only in cell-derived trypomastigotes, detecting two bands of ∼18 kDa by western blot (Figure S3).

## Discussion

Our first goal in this work was the identification of genes preferentially expressed in the trypomastigote stage of *Trypanosoma cruzi*, the etiological agent of Chagas' disease. To achieve this goal we followed an approach based on the sequencing of a subtractive cDNA library. Most of the clones of this TcT-E library represent mRNAs that are preferentially expressed in trypomastigotes, as confirmed by northern and reverse northern blots (Fig 1). The sequence information derived from the TcT-E library allowed us to identify genes that were not previously described in *T. cruzi*. For example, we found several clones with similarity to proteins that have been proposed to function in processes such as rRNA processing, ribosome assembly and the control of cell cycle in other eukaryotic organisms (Bop1, Nop56, BEM, Cwf17; see Tables S1 and S2) [31], [32], [33], [34]. Little is known in *T. cruzi* about these checkpoints in the cell cycle and it is interesting to note that the preferential expression of these mRNAs was detected in a non-replicative stage of the parasite. The lack of transcriptional control in trypanosomatids is well known, and, therefore, stage-specific differences in mRNA abundance are likely to be the result of selective mRNA stabilization and/or the absence of degradation mechanisms for those transcripts [22]. Therefore, one possibility is that these transcripts are being accumulated for the production of the corresponding proteins once the trypomastigote differentiates into the replicating amastigote within the host cell, or when the trypomastigote differentiates into epimastigotes upon entering the insect vector.

After clustering the TcT-E dataset we identified a sequence that was found to be over-represented in trypomastigotes and that has a subregion of 100 bp with high similarity to the 3′ UTR of the *T. cruzi* flagellar antigen FL-160-2. This observation called our attention because the FL-160-2 gene is a member of a numerous family that is differentially expressed on the surface of trypomastigotes and is involved in parasite virulence [19], [20]. All these facts suggested that the 100 bp region could be part of a longer conserved region, and, indeed, we reconstructed a 280 bp element by multiple sequence alignment of TcT-E clones that matched the 100-bp motif that we named TcT-E *element* (TcT-E*elem*), because of its high representation in the TcT-E library. Although we ended up associating the TcT-E*elem* with the 3′ UTR of the new TcTASV family, we also observed that part of the TcT-E*elem* (∼120–150 nt) is also found downstream of genes that do not belong to this family. For example, some hypothetical proteins, trans-sialidase genes or other coding sequences harbouring part of the TcT-E*elem* were identified (Tc00.1047053507875.70, Tc00.1047053504533.40, Tc00.1047053507491.20). Interestingly, in all those cases, the 120–150-bp subregion of the TcT-E*elem* is farther downstream from the stop codon than in the case of TcTASVs genes. Post-transcriptional cis-acting elements conserved among different genes and included into more extended 3′ UTRs have been previously identified. The existence of a regulatory region of 770 bp that is specific for amastin genes and that contains a 450-bp zone shared by amastin and other developmentally-regulated mRNAs has been reported in *Leishmania* [35]. This 450-bp sub-region (currently known to be part of the LmSIDER1 subfamily) mediates the translational regulation of mature transcripts in response to elevated temperature, the main environmental change that the parasite encounters upon its transmission from the vector to the mammalian host [36], [37]. Taking this into account, it could be hypothesized that a general stage-specific regulation of genes can be achieved in a similar way in *T. cruzi*. The 120–150-bp motif that is shared by different genes preferentially expressed in trypomastigotes, such as FL-160, TS and TcTASVs, could be involved in this stage-specific expression, probably forming part of a post-transcriptional regulon that allows the coordinated expression of these genes [38].

The new TcTASV gene family described in this work is composed of 38 members in the CL-Brener strain, none of which show significant similarity to other surface proteins in *T. cruzi*. A meticulous comparative analysis between sequences of TcTASV, MASPs, TcMUCII and mucin-like genes, shows that each of the families diverge in a diferent branch of the computed phylogenetic tree, thus reinforcing the idea that these are indeed different protein families.

After the recent re-assembly of the genome of *T. cruzi* [5], previously annotated genes that we have now identified as TcTASVs could be found in 5 chromosomes, with a high proportion of TcTASVs-A on chromosome 16 and almost all annotated TcTASV-Cs on chromosome 24. TcTASV genes are apparently not arranged in tandem and most of them are surrounded by other hypothetical proteins (they are not TcTASVs). However, the majority of the TcTASV genes were not annotated by the genome-sequencing consortium and are still left out of the final genome assembly (they are only present as ORFs identified in unassembled or small partially assembled contigs). This is highly suggestive of assembly problems that occur frequently when highly similar genes are present in a moderate to high copy numbers. Therefore, it is possible that the copy number of TcTASVs genes in the CL-Brener genome could have been underestimated because of the collapse of repeated genes into fewer copies during assembly. However, the identification of a similar number of members of the TcTASV family in another type II strain (RA) of *T. cruzi* probably indicates that for lineage II the number of TcTASV genes is around 45, whereas those for lineage I is probably around 30, i.e., the lowest number. These conclusions were derived from PCR experiments using oligonucleotides designed on highly conserved regions. Therefore, there is still the possibility that other TcTASV genes could not be amplified by these primers.

Complex glycoproteins cover the surface of all the developmental stages of trypanosomatid human pathogens [2], [39]. Among the species-specific families, the best-studied ones are probably the mucins of *T. cruzi* and the proteophosphoglycans (PPGs) of *Leishmania spp*. Both protein families are rich in Ser, Thr and Pro residues, are retained in the membrane by GPI anchors and can be released from the parasite. In the case of TcTASVs, the amino acid composition is different, being enriched in Ala, Ser and Val. Regarding their expression, different groups of mucins and proteophosphoglycans are developmentally expressed, i.e. TcMUC I and II are expressed in the mammalian stages of *T. cruzi*, whereas TcSMUGs are only found in insect-derived stages ([40] and reviewed in [41], [42], [43]). In *Leishmania*, filamentous PPGs are secreted by promastigotes and have been implicated in protection from digestive enzymes in the insect midgut and in the formation of a plug in the sandfly digestive tract, which causes an increased frequency of feeding and correlates with parasite invasion and virulence [44], [45], [46]. On the other hand, membrane-bound PPGs have been implicated in parasite binding and invasion of macrophages [47], [48], [49]. For both mucins and PPGs several mechanisms leading to immune system evasion have also been demonstrated [41], [42], [49], [50], [51], [52], [53]. Therefore, it is clear that the parasite expresses different kind of mucins or PPGs, even with a differential cellular localization, in the different developmental stages in order to invade and persist in the parasitized host. In this work we demonstrate that TcTASV-A are expressed in trypomastigotes and could not be detected in other stages, suggesting that the TcTASV population could undergo developmental regulation. However, we cannot completely rule out the possible expression in other parasite stages, because we did not analyze the expression of the TcTASV-B nor TcTASV-C subfamilies and used only one peptide to obtain anti-TcTASV-A antibodies. Related to this, after following a proteomic approach Atwood *et al.* were able to identify a peptide in trypomastigote and amastigote extracts that is completely conserved (100% identity) in the TcTASV-A subfamily. The expression of TcTASV-A in amastigotes, though, probably occurs at very low levels since we were unable to detect TcTASV-A proteins in this parasite stage. Moreover, only one spectrum was detected by Atwood *et al.* in amastigotes (vs. 5 in trypomastigotes) [28].

Based on computational analyses, we predicted a signal peptide in the amino terminus and a potential site for the addition of a GPI anchor at the carboxy terminus of TcTASVs. However, at this moment, we cannot rule out the possibility that some members of TcTASV have a membrane-associated expression and others a cytosolic or secreted form.

In summary, in the present work we have identified and partially characterized a new surface protein family in *T. cruzi* wich we named TcTASV. All TcTASV members have a conserved 3′ untranslated region (the TcT-E*elem*, also identified for the first time here), conserved amino- and carboxy- termini, and could be grouped into three subfamilies according to the relative molecular mass of the predicted proteins. The presence of a high number of Ser and Thr susceptible to glycosylation as well as a signal peptide and a consensus sequence for the addition of a GPI anchor were predicted. The expression of the TcTASV-A subfamily in trypomastigotes was demonstrated. One other interesting characteristic of the TcTASV family is the lack of orthologues in other trypanosomatids. Finally, we would like to emphasize that TcTASV is a new gene family in *T. cruzi*, which so far had remained unnoticed (unannotated or missing from the assembled genome). We have worked closely with other groups to make sure that this is solved in future releases of *T. cruzi* genome databases. However, given the still draft nature of the *T. cruzi* genome, the possibility exists that this can happen for other genes. Moreover, by means of a genetic vaccination approach, one of the members of TcTASV (formerly TcYASP) has been found as part of a protective pool of antigens [3], which suggests that they are possible good vaccine candidates.

## Supporting Information

Alternative Language Abstract Translation of the abstract into Spanish by Valeria Tekiel.
(0.02 MB DOC)

Figure S1 An amino acid alignment of all TcTASV and mucin-like genes and ORFs retrieved from TriTrypDB was used to construct a phylogram (unrooted Neighbor-Joining tree). The tree evidences that TcTASV is a novel protein family in *T. cruzi* and different from mucin-like genes. Besides, it is shown that 6 genes that at the time of writing were annotated in TriTrypDB as mucin-like genes are actually members of he TcTASV family. Boostrap values corrsponding to 1000 permutations are shown in the phylogram. Blue hexagons indicate the genes were annotated as mucin-like in TriTrypDB.
(0.46 MB TIF)

Figure S2 Unrooted Neighbor-Joining tree analyzing the relationshop between different *T. cruzi* glycoprotein families. The phylogram tree was derived from multiple sequence alignments between sequences of TcTASV (n = 15), MASPs (n = 15; orange rectangles), TcMUCII (n = 15; pink circles) and mucin-like (n = 15; blue hexagons) genes. Each of the families is confined to diferent branches of the tree, all with high bootstrap values, thus reinforcing the idea that they are different protein families. Boostrap values correspond to 1000 replicates.
(0.50 MB TIF)

Figure S3 Western blot (12% gel) of total protein extracts (15 µg) from CL-Brener trypomastigotes using affinity-purified anti-TcTASV-A antibodies.
(0.70 MB TIF)

Table S1 BLAST reports for all TcT-E clones searched against trypanosomatid ESTs and public protein databases (supplementary online material available at http://genoma.unsam.edu.ar/projects/tct-e/tct-e.p.html). The TcT-E clones are ordered alphabetically and according to their pattern of hits against the different databases searched. The coding status (coding/non-coding/indeterminate) is also provided based on predictions using testcode [54].
(0.05 MB DOC)

Table S2 Table of TcT-E clones with homology to SwissProt and nr databases (BLASTX results). A list of the 50 best hits against SP and nr is provided.
(0.09 MB XLS)

Table S3 The information of the TcT-E clustered dataset. The sequence of all clusters and contigs and a list of the TcT-E EST clones that belong to each contig is provided.
(0.05 MB PDF)

Table S4 Table showing the BLAST results of each of the TcTASV members experimentally identified in this work against the *T. cruzi* genome database (BLASTP vs. protein and ORFs databases at http://TriTrypDB.org). Table S4 includes the best hit found for each TcTASV, the GenBank accession number of the TcTASVs, the protein and contig identifiers according to GeneDB/TriTrypDB, the BLAST expect value, and other observations such as whether the protein was correctly annotated or not.
(0.01 MB XLS)

Table S5 *In silico T. cruzi* proteome and orfeome survey for the identification of additional TcTASV members. The *T. cruzi* database was searched for additional TcTASV members by using experimentally identified TcTASV-A, TcTASV-B and TcTASV-C sequences as queries. The IDs of all ORFs that we identified as belonging to the TcTASV family (including sub-family) are listed. Also, the gene ID (when annotated), the position of the identified ORF in the contig, the presence of the TcT-E element at 30–70 bp from the stop codon and other observations are indicated.
(0.02 MB XLS)
