# Supplementary material for: TcTASV: A Novel Protein Family in Trypanosoma cruzi Identified from a Subtractive Trypomastigote cDNA Library
Source: PLoS Negl Trop Dis. 2010 Oct 5;4(10):e841. doi: 10.1371/journal.pntd.0000841 (PMC2950142; doi:10.1371/journal.pntd.0000841)
Supplement: Alternative Language Abstract — Translation of the abstract into Spanish by Valeria Tekiel. (0.02 MB DOC) [file pntd.0000841.s001.doc]

### Alternative Language Abstract

### Translation of the abstract into spanish by Valeria Tekiel.

### Resumen

**Antecedentes:** La identificación y caracterización de antígenos expresados en los estadios de *Trypanosoma cruzi* que parasitan al huésped mamífero es crucial para el desarrollo de vacunas y pruebas diagnósticas. Los genes que se expresan preferencialmente en el estadio tripomastigote muy probablemente estén involucrados en procesos específicos de este estadio parasitario, como por ejemplo invasión celular y evasión de la respuesta inmune.

**Métodos/ Hallazgos principales:** Con el objetivo inicial de indentificar ESTs específicos del estadio tripomastigote, construímos y secuenciamos una biblioteca de ADNc de tripomastigotes substraída con ADNc de epimastigotes (biblioteca TcT-E). Más del 45% de los clones secuenciados no presentaron identidad con ARNm o proteínas anotadas previamente en bases de datos. La existencia de estos transcriptos fue confirmada mediante ensayos de *northern* blot y *northern* blot reverso, proveyendo nueva información acerca de la expresión de esos ARNm en el estadio tripomastigote de *T. cruzi*. Por otro lado, luego de agrupar por *clustering* el set de datos derivado de la secuenciación de la biblioteca TcT-E, identificamos un elemento con secuencia conservada de 280 pb (TcT-E*elem*, por elemento TcT-E) que posteriormente mapeamos en la región 3’ no traducida (UTR, por sus siglas en inglés: untranslated region) de diferentes marcos abiertos de lectura (ORFs, por la iniciales en inglés: open reading frame). Mediante RT-PCR y secuenciación automática, identificamos diferentes ARNm maduros conteniendo el TcT-E*elem* en la región 3’ UTR. Las proteínas codificadas por los ORFs río arriba del TcT-E*elem* resultaron ser miembros de una nueva familia de proteínas de superficie en *T. cruzi*, que denominamos TcTASV debido a su expresión diferencial en el estadio tripomastigote y su composición rica en alaninas, serinas y valinas. Las caracterísicas distintivas de la familia TcTASV son regiones amino y carboxilo terminales conservadas entre todos sus miembros, y una región central variable (en secuencia y longitud) que permite definir tres subfamilias TcTASV (A, B y C). La inspección detallada de las secuencias depositadas en la base de datos de *T. cruzi* (ORFs y genes), nos permitió determinar que la familia TcTASV está compuesta por 38 genes/ORFs en la cepa CL-Brener (linaje II). Ya que la familia TcTASV no posee ortólogos en otros kinetoplástidos, investigamos si TcTASV estaba presente en otras cepas de *T. cruzi* que no hubieran sido secuenciadas hasta el momento. Identificamos 48 genes TcTASV en la cepa RA (linaje II) y 28 miembros en la cepa Dm28 (linaje I), estableciendo que la familia TcTASV está conservada en ambos linajes parasitarios. También determinamos –mediante análisis filogenéticos detallados- que la familia TcTASV es diferente de otras familias multigénicas de *T. cruzi* (TcMUCII, mucin-like, MASP) que poseen una estructura similar.

**Significancia/Conclusiones:** En este trabajo identificamos y caracterizamos parcialmente una nueva familia de proteínas de superficie en *T. cruzi*: TcTASV.
