# Supplementary figures and images for: TcTASV: A Novel Protein Family in Trypanosoma cruzi Identified from a Subtractive Trypomastigote cDNA Library
Source: PLoS Negl Trop Dis. 2010 Oct 5;4(10):e841. doi: 10.1371/journal.pntd.0000841 (PMC2950142; doi:10.1371/journal.pntd.0000841)

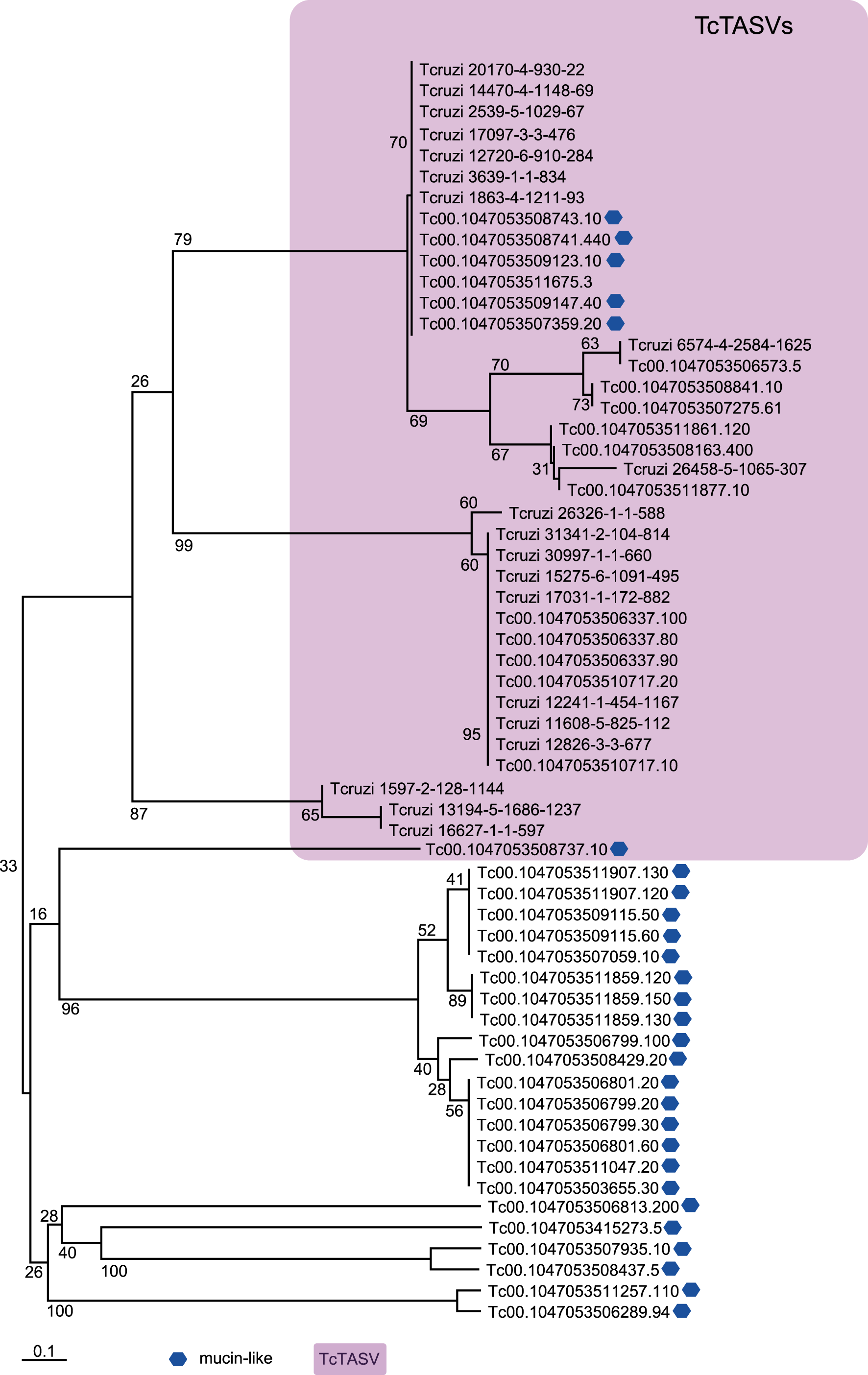

Supplement: Figure S1 — An amino acid alignment of all TcTASV and mucin-like genes and ORFs retrieved from TriTrypDB was used to construct a phylogram (unrooted Neighbor-Joining tree). The tree evidences that TcTASV is a novel protein family in T. cruzi and different from mucin-like genes. Besides, it is shown that 6 genes that at the time of writing were annotated in TriTrypDB as mucin-like genes are actually members of he TcTASV family. Boostrap values corrsponding to 1000 permutations are shown in the phylogram. Blue hexagons indicate the genes were annotated as mucin-like in TriTrypDB. (0.46 MB TIF) [file pntd.0000841.s002.tif]

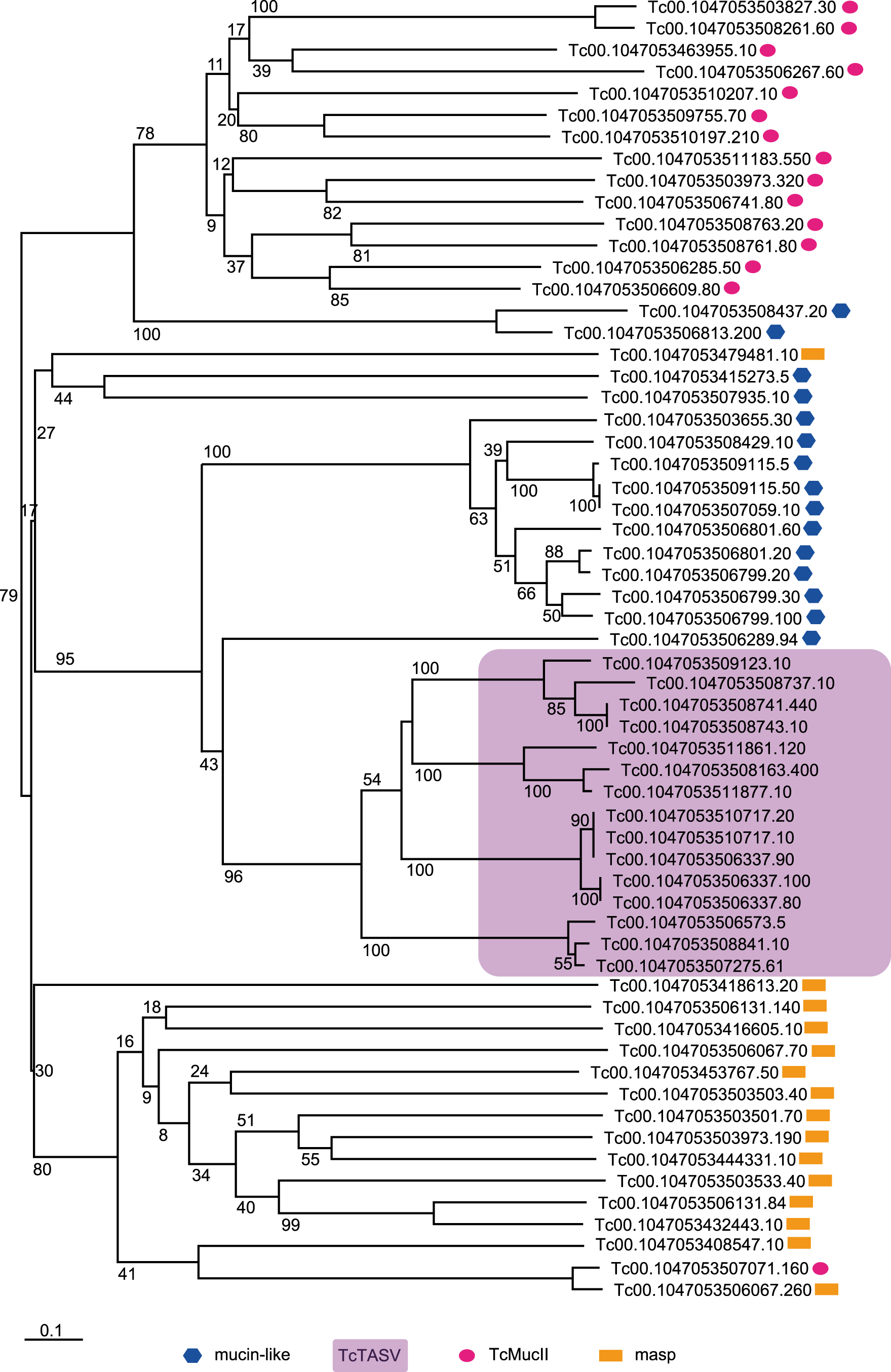

Supplement: Figure S2 — Unrooted Neighbor-Joining tree analyzing the relationshop between different T. cruzi glycoprotein families. The phylogram tree was derived from multiple sequence alignments between sequences of TcTASV (n = 15), MASPs (n = 15; orange rectangles), TcMUCII (n = 15; pink circles) and mucin-like (n = 15; blue hexagons) genes. Each of the families is confined to diferent branches of the tree, all with high bootstrap values, thus reinforcing the idea that they are different protein families. Boostrap values correspond to 1000 replicates. (0.50 MB TIF) [file pntd.0000841.s003.tif]

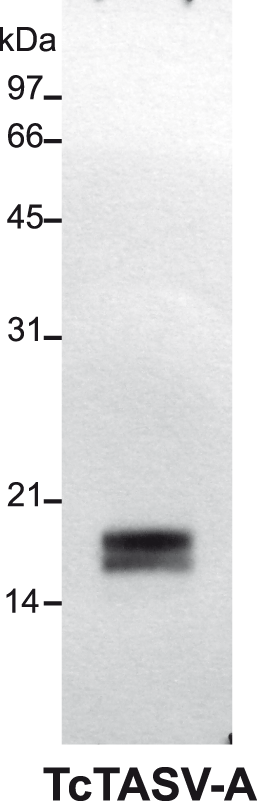

Supplement: Figure S3 — Western blot (12% gel) of total protein extracts (15 µg) from CL-Brener trypomastigotes using affinity-purified anti-TcTASV-A antibodies. (0.70 MB TIF) [file pntd.0000841.s004.tif]
