# Supplementary material for: TcTASV: A Novel Protein Family in Trypanosoma cruzi Identified from a Subtractive Trypomastigote cDNA Library
Source: PLoS Negl Trop Dis. 2010 Oct 5;4(10):e841. doi: 10.1371/journal.pntd.0000841 (PMC2950142; doi:10.1371/journal.pntd.0000841)
Supplement: Table S1 — BLAST reports for all TcT-E clones searched against trypanosomatid ESTs and public protein databases (supplementary online material available at http://genoma.unsam.edu.ar/projects/tct-e/tct-e.p.html). The TcT-E clones are ordered alphabetically and according to their pattern of hits against the different databases searched. The coding status (coding/non-coding/indeterminate) is also provided based on predictions using testcode [54]. (0.05 MB DOC) [file pntd.0000841.s005.doc]

**Supporting Information Table S1.** BLAST reports for all sequence similarity searches using TcT-E clones against trypanosomatid EST and other protein databases are available as supporting online material available at <http://genoma.unsam.edu.ar/projects/tct-e/tct-e.p.html>. The TcT-E clones are ordered alphabetically and according to their pattern of hits against the different databases searched. The coding status (coding/ non-coding/ indeterminate) is also provided based on predictions using testcode [54].

**Please, refer to the URL** <http://genoma.unsam.edu.ar/projects/tct-e/tct-e.p.html> to find the complete and detailed information about TcT-E library clones and BLAST results.

**Sequence similarity summaries sorted by database matched.**

A summary of the results of different sequence similarity searches is available as a clickable table (see below and at the website mentioned above). The table highlights the pattern of hits against the databases searched. The pattern is a graphical representation of a significant hit (+) or a non-significant hit/no-hit (-).

**Sequence similarity summaries by clone.**

These summaries are also available [sorted by name (alphabetically).](http://genoma.unsam.edu.ar/projects/tct-e/tca-c.html)

**In the following table, the databases have been encoded as follows:**
S = Swissprot (Protein)
N = nr, GenBank (Protein)
E = T. cruzi epimastigotes ESTs
K = ESTs from all kinetoplastid parasites. This dataset contains sequences in E
T = T. cruzi trypomastigote ESTs (A. Gonzalez)

| **S** | **N** | **E** | **K** | **T** | **Number** |
| --- | --- | --- | --- | --- | --- |
| + | + | + | + | + | 2 |
| + | + | + | + | – | 3 |
| – | + | + | + | + | 1 |
| + | – | + | + | + | 1 |
| – | + | + | + | – | 14 |
| – | – | + | + | + | 31 |
| – | – | + | + | – | 102 |
| + | + | – | – | – | 14 |
| – | + | – | – | – | 32 |
| + | – | – | – | – | 1 |
| – | – | – | – | + | 14 |
| – | – | – | – | – | 188 |
