# Supplementary material for: TcTASV: A Novel Protein Family in Trypanosoma cruzi Identified from a Subtractive Trypomastigote cDNA Library
Source: PLoS Negl Trop Dis. 2010 Oct 5;4(10):e841. doi: 10.1371/journal.pntd.0000841 (PMC2950142; doi:10.1371/journal.pntd.0000841)
Supplement: Table S3 — The information of the TcT-E clustered dataset. The sequence of all clusters and contigs and a list of the TcT-E EST clones that belong to each contig is provided. (0.05 MB PDF) [file pntd.0000841.s007.pdf]

>cl1ct1cn1 COVERAGE:1.0; CRAWID: 1; TOTAL_ESTS:1; ESTS:vale49384; LENGTH:
410bp; MAP: ; ALT_CONSENSI:0
TATTAGCGTGGTCGTGGTTCGAGGTACAAGCTTTTTTTTTTTTTTTTTTTTTTTTTTTTT
TTTACCAAAATTATTTAAGGGAACAAAAAAGTAAGAATGAGAATCCCGCAAGACTCCCGG
AAAACAATTAAGAAAAAAGAAAAAAGAAAAAAAATTGTTTCGGCAGGAATTTCATGTGCC
ATGCGGCTCAAGACGATCGGGGGCGTGCATATATATTACCTGCCCAGACGAAGAATGGAA
TAATAATAATAATATTGCATTCTTCCACTAGCCCACACAACATTATTCGCATTTTATGCG
GGATCCGGCCTTTTTTTCCAATCCTCGTTCGTGCAGTCCGGGGGAAGCACAACCGCAGGG
CCGGCACCGCGTGTGGGTGACGAAGCAAATTAAAAAAAAAAAAAAGGAAAA
>cl1ct2cn2 COVERAGE:0.95; CRAWID: 1; TOTAL_ESTS:5; ESTS:
vale49161,vale49167,vale49131,vale49569,vale49526; LENGTH:461bp; MAP: ;
ALT_CONSENSI:0
GGCCGGCATGGCGGGCGCCCGGGTGTACTTGCGGCCTGCCGCCCGGGCAGGTTTTTTTTT
TATCAAAGAAAAAAGAAAAGCCAAAAAAGAAAGAATGAGAATCCCGCAGGGCTCCCGGAA
AGCAAAAAAGAAAAAAGAAAAAAAAAGAAAAAAAAAAGAAAAAAAAGAAATGATTCGGCA
GTAATTTCATGTGCCATGCGGCTCAAGACGATCGGGTGCGTGCATATATATTACCTGCAC
AAAAGAAAAATGAAATAATAATAATAATAACATCGCGTTCTTCCACTAACCCACACAACA
TTCTTCGCATTTCATGCGGCATCCGGCCTTTTCTTCCAATCCTCGTTCGTGCAGTCCGTG
GGACGCACAGCCGCAGGGCCGGCACCGCGTGTGGGTGACAAAGCAAATTTAAAAAAAAAA
AAGGAAAACACACATACTGTACCTCGGCCGCGACAACACTA
>cl1ct3cn3 COVERAGE:0.93; CRAWID: 1; TOTAL_ESTS:6; ESTS:
vale49246,vale49347,vale49502,vale49369,vale49521,vale49618; LENGTH:650bp;
MAP: ; ALT_CONSENSI:0
TTCGAGCGGCCGTCCGTGCAGGTCGAACACGTGGTTTTTTGTCCCGAAGATTCCAGTCAG
ACGCCCTCTTCTCTATCTGGAGGCACCGCCACTGGGATGCGGGATCCCGGCGGnGCGGAC
AGCAGCGACGCCGCCACTGTGTGGGTGCGCATGTCTCTGCTGCTCGTCACCGCCCTTATC
TGCGCTGCTGTGCGGTGATTGGGATTAGTCGTGCATTTCCTTGCGGGGACGGCTCGTACT
TTATATGTGTTTCCTTTTTTTTTTCTTTTTCTTTTGTGCTTTTGTTTTTGACCACACGCGG
TGCCGGCCCTGCGGCCGTGCGTCCCACGGACTGCACGAAAGGGGGATTGGAGGAACAGGC
GGGATGCCGCATGAAATGTGAGGGATGTTGTGTGGGCTGGTGGAATAATGCGATCTTTTT
TTATTATTACGTTTTTCGTTTGTGCAGGTAATATGTGCAAGCACCCAATCGTCTTTAGCT
GCATGGCACATGAAAGTCCTGCCGAATCATTTTTTTTTCCATGAGTCCTGCAGGGGACCC
ACATTTTTTTTGGTTTGTTTGTTTCATTCTTTTTCATTTTTGGCAAAAAAAAAAAAAAAAA
AAAAAAACCGGAAAAACAAAAAAAAAGCTTGTACCTGCTCGGGCGGCCGC
>cl1ct4cn4 COVERAGE:0.91; CRAWID: 1; TOTAL_ESTS:8; ESTS:
vale49158,vale49200,vale49317,vale49351,vale49349,vale49379,vale49386,vale4
9614; LENGTH:485bp; MAP: ; ALT_CONSENSI:0
GACTCATGAAAAAAAAAATAAATGATTCGGCAGGACTTTCATGTGCCATGCAGCTAAAGA
CGATTGGGTGCTTGCACATATTACCTGCACAAACGAAAAACGTAATAATAAAAAAGAGCT
CGCATTATTCCAGCATCCCACACAACATTCTTCACATTTCATGCGGCATCCGGCCTGCTC
CTCCAATCCTCCTTTCGTGCAGTCCGTGGGACGCACAGCCGCAGGGCCGGCAACGCGTGT
GGTCAAAAACAAAAGCACAAAAAAAAAAAAGAAAAAAAAAAGGAAACACATATAAAGTAC
GAGCCATCCCCGCAAGGAAATGCACGAGTAATCCTAATCACCGCACAGCAGCACAGACAA
GGGCGATGAAGAGCAGCAGCAGGGGCGTGCGCACCCACACAGTGGTGACATCGCTGCCGT
CCGGACCGACAAAATTCTGCAACAAAGCGGCGATATTTTCTGTACCTGCCCGGGCGGCCG
CTTGA
>cl1ct5cn5 COVERAGE:0.94; CRAWID: 1; TOTAL_ESTS:17; ESTS:
vale49160,vale49149,vale49135,vale49267,vale49559,vale49214,vale49307,vale4
9309,vale49327,vale49360,vale49601,vale49597,vale49570,vale49503,vale49525,
vale49616,vale49580; LENGTH:709bp; MAP: ; ALT_CONSENSI:0

CCGGGCAGGTACAGTATGTGTGTTTTCCTTTTTTTTTTTTTAAATTTGCTTTGTCACCCA
CACGCGGTGCCGGCCCTGCGGCTGTGCGTCCCACGGACTGCACGAACGAGGATTGGAAGA
AAAGGCCGGATGCCGCATGAAATGCGAAGAATGTTGTGTGGGTTAGTGGAAGAACGCGAT
GTTATTATTATTATTATTTCATTTTTCTTTTGTGCAGGTAATATATATGCACGCACCCGA
TCGTCTTGAGCCGCATGGCACATGAAATTCCTGCCGAATCATTTTCTTTTTTTTTTCTTT
TTTCTTTTTTGCTTTCCGGGAGCCCTGCGGGATTCTCATTCTTTCTTTTTTGTCTTTTCT
TTTTTCTTTGGGTAAAAAAAAAAAAAAAAAAAAAAAAAACTATAAAAGGCTTACAGGTAA
AAGAACAGCAATGAAGTAAAAAGTAAAAAAAAACACAAAACAAAGAGCTGCACAGTATGG
CTTCAGCGCGCACGCACCCATCCTCAAATGTGATTCCACGCAAACAGTTGTGTGCGTGCC
ATGCACATGAGTGTGTCTGGGATGCGTTCAACGTGACGGATGCGGGGAGCGGCAAGGAGC
ACACAGCAGGGCAGCGAATGCAAATAAAAACAGTAAAACACAATCATCATTTTTTTCGTA
TTTATTACTTAGGAAAACAATAAACGACCTGCCCGGGCGGCCGCTCGAA
>cl1ct6cn6 COVERAGE:0.94; CRAWID: 1; TOTAL_ESTS:19; ESTS:
vale49556,vale49299,vale49192,vale49290,vale49311,vale49506,vale49357,vale4
9366,vale49599,vale49606,vale49359,vale49378,vale49584,vale49571,vale49515,
vale49517,vale49529,vale49610,vale49579; LENGTH:454bp; MAP: ; ALT_CONSENSI:
0
TAGCGTGGTCGCGTCGCCGGGGCAAGCTTTTTTTTTTTTTTTTTTTTTTTTTTTTTTTTT
ACCAAAAACAGAAAAGGAAACAAAAAAGAAAGAATGAGAATCCCGCAGGACCCCCGGAAA
ACAATAAAGAAAAAAGAAAAAAGAAAAAAGAAAAAAAAATTGATTCGGCAGGAATTTCATG
TGCCATGCGGCTCAAGACGATCGGGTGCGTGCATATATATTACCTGCACAGACGAAGAAT
GGAATAATAATAATAATATCGCATTCTTCCACTAGCCCACACAACATTATTCGCATTTCA
TGCGGCATCCGGCCTTTTCTTCCAATCCTCGTTCGTGCAGTCCGTGGGAAGCACAGCCGC
AGGGCCGGCACCGCGTGTGGGTGACGAAGCAAATTAAAAAAAAAAAAAGAAAAGGAAAAC
ACACATACTGTACCTGCCCGGGAAGCCGCTCGAA
>cl1ct7cn7 COVERAGE:0.95; CRAWID: 1; TOTAL_ESTS:21; ESTS:
vale49153,vale49209,vale49145,vale49123,vale49250,vale49172,vale49183,vale4
9261,vale49195,vale49288,vale49298,vale49206,vale49301,vale49237,vale49338,
vale49329,vale49585,vale49326,vale49363,vale49519,vale49524; LENGTH:574bp;
MAP: ; ALT_CONSENSI:1
TAGCGTGGTCGCGGCCGTCGCCGCTTTGTTGCCGACTTTCAGTCCGGACGGCAGCGACGT
CACCACTGTGTGGGTGCGCACGCCCCTGCTGCTGCTCGTCATCGCCCTTGTCTGTGCTGC
TGTGCGGTGATTGGGACTAGTCGTGCGTGTCCTTGCGGGGACGGCTAGTACAGTATGTGT
GTTTTCCTTTTTTTTCTCTTCTTTTTTTAATTTGCTTTGTCACCCACACGCGGTGCCGGC
CCTGCGGCTGTGCGTCCCACGGACTGCACGAGCGAGGATTGGAAGAAAAGGCCGGATGCC
GCATGAAATGCGAGGAATGTTGTGTGGGCTAGTGGAAGAACGCGATGTTATTATTATTAT
TTTATTTTTCTTTTGTGCAGGTAATATATATGCACGCACCCGATCGTCTTGAGCCGCATG
GCACATGAAATTCCTGCCGAATCATTTCTTTTCTTTTTTCTGTTTTCCGGGAGTCCTGCG
GGATTCTCATTCTTTTTTTCGTTTCATTTCCTGTTTTGGTTAAAAAAAAAAAAAAAAAAA
AAAAAAAAGCTTGACCTCCCCGGGCGGCCGCCTA
>cl1ct8cn9 COVERAGE:0.93; CRAWID: 1; TOTAL_ESTS:23; ESTS:
vale49154,vale49120,vale49201,vale49222,vale49126,vale49240,vale49129,vale4
9127,vale49181,vale49173,vale49258,vale49277,vale49306,vale49225,vale49348,
vale49364,vale49600,vale49361,vale49344,vale49350,vale49534,vale49536,vale4
9622; LENGTH:458bp; MAP: ; ALT_CONSENSI:0
TAGCGTGGTCGCGGCCGGGGTGCTAACGCCTGTTGTTTTTTTTGTTTTTTTTTTTTTTTA
CCAAAGAAAAAAGAAAAGACAAAAAAGAAAGAATGAGAATCCCGCAGGGCTCCCGGAAAG
CAAAAAAGAAAAAAGAAAAAAAAAAGAAAAAAAAAAAGAAAAAAAAGAAATGATTCGGCA
GGAATTTCATGTGCCATGCGGCTCAAGACGATCGGGTGCGTGCATATATATTACCTGCAC
AAAAGAAAAATGAAATAATAATAACATCGCGTTCTTCCACTAGCCCACACAACATTCTTC

GCATTTCATGCGGCATCCGGCCTTTTCTTCCAATCCTCGTTCGTGCAGTCCGTGGGACGC
ACAGCCGCAGGGCCGGCACCGCGTGTGGGTGACAAAGCAAATTTAAAAAAAAAAAAAAGG
AAAACACACATACTGTACCTGCCCGGGCGCCCGCTCGA
>cl2ct9cn10 COVERAGE:1.0; CRAWID: 1; TOTAL_ESTS:1; ESTS:vale49501; LENGTH: 478bp; MAP: ; ALT_CONSENSI:0
TTTTGCGGCCAGGACTGGCAGACACCCGCATTTTTTTACACACACACAGCCACACCCTGC
CGGGTTCTAGGGATTTTTTTGCTTTTTGTGTGCATTTCACGCGCTGGCAGTGCCGTCAGC
ATTTCTCTTTTCCCCGTGATGATTTTTTTTTTTTGCTGCGCTCAATTACCTCCACACACA
CACGAGTGACTGAGCTCTGCGGGCCGCACTCACGCGCCGATGAAAAGGCTGAGGGGAGCA
GCAACACGCAGATGGCCCCATGAAATATAAACGATGTGCGGACACATGGAGGTCGGACGG
TTGAAAGACATGCAACTATTTAATTTTATTTTGCGGGCAAAATATATGCAGACGGTTCGG
TGTGGCCACACGGCAGCCAGGCGAATTTATTATTATTATGATTGCTTTTTTTTTTATCAA
TTTTAAATTTGTGTGGAACAGTAATATAGGAAGTTCTTTTTTTTTTTTTTATGACGGG
>cl2ct10cn11 COVERAGE:1.0; CRAWID: 1; TOTAL_ESTS:1; ESTS:vale49155; LENGTH: 410bp; MAP: ; ALT_CONSENSI:0
ACGATGCTTGCGGCCGGCACTGGAGGGCGGGGGAATTAAATTTATAGCGGCCGCCCGGGC
AGGTCGGACTCTTTTCATGTCTGCGTGCTTCTCTCAGTCCCCACACTCACACGAGTGAC
TGTGCTCTGCAGGCCGAACTCACGCGCCGATGAGAGGGCTGAGGGGAGCACCAACACGCA
GATGACCCCATGAAATATAAACGATGTGCGGACACATGGAGGGTCAGACGGTTGAAAGAC
ATTTAACTATTTAATTTTTAATTTGCGGGCAAAATATACAAACAGTTTGGTAAGAGCGCA
CGGCAAACAGGTGAATTTTTTTTGTGTGGCTTCTCTTTTCTTTTTTTTTCATTGGGTTGA
GTGGAACAGTTATAGAGGACGACTTTTTTTGTTTTTTAAATAATTATTTT
>cl2ct11cn12 COVERAGE:1.0; CRAWID: 1; TOTAL_ESTS:1; ESTS:vale49122; LENGTH: 401bp; MAP: ; ALT_CONSENSI:0
TTCGAGCGGTCTCCCGGGCAGGTGTCATGTTGGTGTGCTTCTCTCAGTCCCCACACACAA
GAAGAGTGACTGTGCTCTGCGGGCCGCACTCACGCGCCGATGGGAGGGCTGAGGGGAGCA
GCAACACGTGGATGACCCCATGAAATATGAACGATGTGCGGACACACAGAGGGTCGGACG
GTTGAAAGACATTTAAATATATTTTAAATTTTTTTGTGGTCAAAATATATGCAGACAATT
TCTGGAGCAACATGGTGGTTGGGTGCTTTCTTTGTGTGGATTTTCATTTTTTCTTTTTTT
CTTTTCAAAGTGTGAGGTGTGGAAGGTGAAGATGAATTTTTTTTTTAATTTTAATAATTT
TTTACGAAAAAAAAAAAAAAACCTCGGCCGCGACCACGCTA
>cl2ct12cn13 COVERAGE:1.0; CRAWID: 1; TOTAL_ESTS:1; ESTS:vale49132; LENGTH: 501bp; MAP: ; ALT_CONSENSI:0
GACACCCGCAGCGCCACACACACACAGCTCTACCCTGCCGCCGTGTTTTTAAGATTTTTG
CTGCGCTCACTCTCCCGATGGAGTGACGCGACTCCCGTCATGTCTGCGTGCTTCTCTCAG
CCCCCACACACGAGTGACTGTGCTCTGCGGGCCGCACTCGCACGCCGATGAGAGGGCTGA
GGGGAGCACCAACACGCAGATGACCCCATGAAATATAAACAATGTGCGGACAGACAGAGG
GTCAGACGGCTGAAAGACACGTAAATATCTTTTAATTTTATTTTCGGACAAAATAAAAAA
CAGTTTGGTATGACCACACAGTAACCGGGTGATTTTTGTTTTCTTGTGTGTAGAACAGTA
ATATAAAACGACTTTTTTTTGGTGTGTGTGAAACAGTAGTATAAAACGATTTTTTTTTTG
TTTGTGGAACAGTAGTAGAAAACAACCTTTCTCTTTTGTTTTTTTTGGGTGTGGAACAAT
GGTGGAGGAAATCTTTTTTTT
>cl2ct13cn14 COVERAGE:1.0; CRAWID: 1; TOTAL_ESTS:1; ESTS:vale49275; LENGTH: 382bp; MAP: ; ALT_CONSENSI:0
TTCGAGCGGCCGCCCGGGCAGGTCTCCCGATGGAGTGACGCGACTCCCGTCATGTCTGCG
TGCCTCTCTCAGTCTCCACACACACGAGTGACTGTGCTCTGCGGGCCGCACTCACACGCC
GATGAGAGGGCTGAGGGGAGCAGCAACACGCAGATGACCCCATGAAATATAAACGATGTG
CGGACACATGGAGGTCAGAAGTTTGAAAGACATTTAAATATCTTTTTAATTCTATTTTCG
GACAAAATATGCAGACGATTTCTGGAGCAACATGGTGGTTGGGTGCTTTTTGTGTGTGTT
TCTTCTTTTTCTTTTTTTTCTTTTCTGTTTTTTGTGTGTAGCCGTAATAGAGGACGACTT

TTTTTCAATTTTCTTTTTTTTT
>cl2ct14cn15 COVERAGE:0.99; CRAWID: 1; TOTAL_ESTS:2; ESTS: vale49303,vale49229; LENGTH:562bp; MAP: ; ALT_CONSENSI:0
CTCACTCTCCCGATGGAGAGACGCGACTTTCGTCATGTCTGCGTGCTTCTCTCAGTCCCC
ACACACACGAGTGACTGTGCTCTGCGGGCCGCACTCGCACGCCGATGGGAGGGCTGAGGG
GAGCAGCAACACGCAGATGACCCCATGAAATATAAACGATGTGCGGACACATGGAGGGTC
AGACGGTTGAAGCACACGCAACCATCTTTTCAATTTTCTTTTCCGGGCAAAATATATACA
GACAGTTTGGTGGGCCACACGGCAGCTAGGCGAAATGTTTCTTGTGTGTTAAGTTTTTCT
TTTCCTTTTTTGGGTGTGGAACAATAGTAGTGGACGAATTTTTTTTTTTTTTGTGTGGAA
CGGTAACAAAGGACAACTTTTTTTTCATATAATTATTGTGTGCGAGGGGGGTTAATATAT
GATGAAAGGGAATTGTTTTTTCTAATTATTTTAAATAAATCTTTTATGAATTGCCTTTGC
TTTGTCTGCAGTGAAGTATCATGCCAAAAAAAAAAAAAAAAAAAAAAAAAAAAAAATTGTA
CCTGCCCCGGCAACCGCTCGAA
>cl2ct15cn16 COVERAGE:0.94; CRAWID: 1; TOTAL_ESTS:3; ESTS: vale49215,vale49220,vale49538; LENGTH:539bp; MAP: ; ALT_CONSENSI:0
TTCGAGCGGCTTCCCGGGCAGGTCTCTCCTTAGTGAGTGACGCGACTCCCGTCATGTATG
CGTGATTTTCTCAGTCCCCACACACGAGTGACTGTGCTCTGCGGGCCGCACTCACGCGCC
GATGAAAGGGCTGAGGGGAGCAGCAACACGCAGATGACCCCATGAAATATAAACGATGTG
CGGAAACATGGAGGGTCAGACGGTTGAAAGACATTTAAATATTTAATTTTCTTTTTGGGC
AAAATAAAAAAAAAAATTACAGCTTGGTATGGCCACACGGTAAGCAGCCGCTTTTTTGTTT
TTGTTTTTGTTTGGAACAGTAATAGAGGACGACTTTTTCTTTTTTATTTTTATTTGTGTG
GAACAGTAATAGAGGACGACTTTCTTTTTTTTTTTTTTTTGTGTGTGTGGAACAGTAGTG
GAGGAAATCTTTTTTTTTTTTTCTTTTTTGTGCGTGGGGTTAAAATGTGAGGAACGGGAAA
TTTTTTGTTTTGTTTTCAAAAAAAAAAAAAAAAAAAAAAAAAAAGCTTGTACCTGCCCGG
>cl2ct16cn17 COVERAGE:0.98; CRAWID: 1; TOTAL_ESTS:3; ESTS: vale49137,vale49219,vale49339; LENGTH:577bp; MAP: ; ALT_CONSENSI:0
AAAGTAATATTACTTGCTCTCTTTGACTGTTCTTCTTGTTTTTCGCGTTACTTTTTGCTG
CGCTCACTCTCCCGAGGGAGAGACGCGACTCCCGTCATGTCTGCGTGCTTCTCTCAGTCC
CCACACACACGAGTGACTGTGCTCTGCGGGCCGCACTCACGCACGCCGATGGGAGGGCTG
AGGGGAGCAGCAACACGCAGATGACCCCATGAAATATAAACGGTGTGCGGACACACAGAG
GGTCAGACGGCTGAAAGACATTTAAATATGTTTTAATTTTATTTTCGGACAAAATATATG
CAGACAGCTTGGTGTGGCCACACGGCAGCCAGGCGAAATGTTTCTTTTGTGTGTAGCTTT
TCTTTTCTTTTTTTTTCTTTTTTTGGGTGGAACAGTAGTAGTGGACAATACTTTTTTTTT
CTTTTTGTGTGGAAAAGTAATAAAGGAGAACAATTTTTTTTTTGTGGAACAGTAATAAAG
GACGACTTTTTCTTTCTTTTTATATAATTATTGTGCGCGAGGGGGGCTAATATATGATGA
AAGGGAGTTGTTATTTTTAATTATTTTAAATAAATCT
>cl2ct17cn18 COVERAGE:0.95; CRAWID: 1; TOTAL_ESTS:4; ESTS: vale49268,vale49294,vale49249,vale49332; LENGTH:633bp; MAP: ; ALT_CONSENSI:0
TTCGAGCGGCCGCCCGGGCAGGTGGGCGGTATCATTCATTTCTTCCCTCTTGTTTTGTTT
TGTTTTTTTGCGGCCAGGGCTGGCAGACACCCGCAGCGCCACGCACACAGCCACACCCTG
CCGCCGTGTTTTTTTTTCTTTTTTGTTTTTGTGTTCATTTCACGCGCTGGCAGTCCCGTC
AGCATTTCTTTTCCCCCGATGTTTTGTTTTCTTTTGCTGCGCTCACTCTCCCGATGGAGT
GACGCGACCGCCTTCATGTTTTTTCGTGCTTCTCTCAGTCCCCACACACGAGTGACTGTG
CTCTGCGGGCCGCACTCACGCGCCGATGAGAGGGCTGAGGGGAGCAGCAACACGCAGATG
ACCCCATGAAATATAAACGATGTGCGGACACACGGAGGGTCGGACGGCTGAAAGACATTT
AAATACTTTTTAATTTTTTTTTGTGGGCAAAAAATATATGGACAGTTTGGTGGGCCACAA
GGCAGCCGGCAGCTTTTTAAAATTTGTGTGGAACAGCAATGGACGATTTCTTTTTCATTT
ATAATTATTTTGTGTGATGGGGTTTGTATAAGACGAAGGGGAAAGCTTTTTTTGTGTTTT
GGACAAATTTTGTGTGGATTGCCGCTGCTTGGA

>cl2ct18cn19 COVERAGE:0.91; CRAWID: 1; TOTAL_ESTS:4; ESTS:
vale49224,vale49190,vale49385,vale49387; LENGTH:558bp; MAP: ; ALT_CONSENSI:
0
TTTAAAAAAAGGATTTTGTTTGCTTGGGGTCTTTGCGTCCTGGGCCGCCGGCACGCCCTT
CAGGGTCGGCCTTGTAAGCATTTCTTTCCCTCCTGCTTTTTTTCTTTTGCTGCGCTCACT
CTCCCGATAAAGTGACGCGACTCCCGTCATGTCTGGGTGCTTCTCTCAGTCCCCACACAC
AAGAGTGACTGTGCTTTGCGGGCCACACTCACGCACCAATAAAACGGCTGAGGGGAGCAG
CAGCACGCAGATGACCCCATGAAATATAAACGCTGTGCGGACACACGGAGGGTCGGACGG
TTGAAAGATATTTAAACATTCAATTTTTAATGTGCGGGCAAAATATACAAACAGTTTGGT
GGGCGCACACAGTAACCAGTGAAATTTTTTTTTGTGTGGTATTTCTTTCTTTATTATTAT
TTGTGTGTAGCCGTAATAGAGGACGACATTTTTCTTTTTTTTTTTCTTATTGGTTCCAAA
GTGTGAGGTGTGGAAGGGTGAAGATCATTTTTTAAAAATTTTAATAnCCAAAAAAAAAAA
AAAAAACAAAAGAGGCTT
>cl2ct19cn20 COVERAGE:0.91; CRAWID: 1; TOTAL_ESTS:4; ESTS:
vale49148,vale49242,vale49178,vale49530; LENGTH:689bp; MAP: ; ALT_CONSENSI:
2
CGGTGGGGCGGCGCACACATTTCTTTCCCTCTTGTTTTGTTTTCTTTGCGGCCAGGGCGG
GCAGACACCCGCAGCGCCACACACACAGCCACACCCTGCCGCCGTGTTGTTAAGATTTTT
TGCTTTTCGTGTGCGCCTTAAGCGCCGGCATTTATTTCGGGGTCGGCCTCCTGAGTATTT
CTCTACCCCCCGATGATTATGTTCTATTTTTGCTGCGCTCACTCTCCCGATGGAGTGACG
CGACTCCCGTCATGTCTGCGTGCTTCTCTCAGCCTCCCCACACACACGAGTGACTGTGCT
CTGCGGGCCGCACTCACGCGCCGATGAAAGGGCTGAGGGGAGCAGCAACACGCAGATGAC
CCCATGAAATATAAACGATGTGCGGACACACAGGGAGGGTCAGACGGTTGAAAGACATTT
AAATATCTTTTAATTTTATTTTCGGACAAAATGTATGCAGACAATTTCTGGAGCAACATG
GTAGTTAGGTGCTTTTCTTATTTGCGTGGTTTTTTTTTCCTTTTTTTAATTCTTTTTTCA
TTTTTTTTTTTTTTTTTTTATCATTTGGGTGCAGCAGTAATAGAGGACTTTTTTTCATTTT
TTCATTTTTTCATTTTTTATTTTTCTTTCCAAAGTGTGAGGTGTGGAAGGGTGAAAATGA
ATTTTTTTTAATTTTAATAAATTTTTATG
>cl2ct20cn23 COVERAGE:0.97; CRAWID: 1; TOTAL_ESTS:9; ESTS:
vale49542,vale49165,vale49289,vale49223,vale49328,vale49598,vale49565,vale4
9372,vale49590; LENGTH:597bp; MAP: ; ALT_CONSENSI:0
TATTAGCGTGGTCTTCGCTGCGGCCAGGGCTGGCAGATTTCTCGCAGCGCCACACACACA
CAGCCACACCCTGCCGCCGTGTTTTTAAGATTTTTGCTGCGCTCACTCTCCCGATGGAGT
GACGCGACTCCCGTCATGTCTGCGTGCCTCTCTCAGTCTCCACACACACGAGTGACTGTG
CTCTGCGGGCCGCACTCACGCACCAATAAAACGGCTGAGGGGAGCAGCAAAACGCAGATG
ACCCATGAAATATAAACGATGTGCGGACACACGGAGGTCGGACGGTTGAAAAAGATGTAT
CTTTCTAAATTTACCTTGTGGTCAAAATATATGTAGACAGTTTGGTGGGCCACACGGCAG
CTAGGCGAAATGTTTCTTGTGTGTTTAGTTTTTCTTTTCCTTTTTTGTGTGTGGAACAAT
AGTAGTGGACGAATTTTTTTTTTTTTTGTGTGGAACAGTAATAAAGGACAACTTTTTTTT
CATATAATTATTGTGTGCGAGGGGGGCTAATATATGATGAAAGGGAATTGTTTTTTCTAA
TTATTTTAAATAAATCTTCCATGAACAAAAAAAAAAAAAAAAAAAAAAAAAAAAAGCTT
>cl3ct21cn24 COVERAGE:0.99; CRAWID: 1; TOTAL_ESTS:2; ESTS:
vale49543,vale49150; LENGTH:408bp; MAP: ; ALT_CONSENSI:0
TGTTTAGTCTACGTTTTGATCTTGAGGTAAAGTTTCCCTTTCAACCCATTGGGGATAAAA
ATTTATTTTTTCAAATTGATGCCTTTGAGGTGAATGCGTTTGTAGATGACATTCTGCGCG
TTGTTTTTGCCTACGCGGATCAGCGGCAAAGTGTTCAGGATGAACGGGGTTGTGTGACAG
ACCGGCCCCGTGATGTCATTTTCAGTAGTTTTGAACCTGATATTTGTTTGGCGTTAAAGA
TGAAACAAAGTCGTTTTGATGTTGTGTTTTTGTGCGATACGGAGATGTCGGAGGACTTTA
AGGATTACCGTTGTTTTGGACTTGTAGAAGGGGCACTGCAATTCTCTGTCTTTATGCATC
TTTCCGGTGTTTCTATCCTTGCTGCATCGTTATGTACCTGCCCGGGCG

>cl4ct22cn25 COVERAGE:0.95; CRAWID: 1; TOTAL_ESTS:3; ESTS:
vale49157,vale49166,vale49142; LENGTH:225bp; MAP: ; ALT_CONSENSI:0
TTCCAGCGGCCTCGCCGCCCAGGTACTGAGGTGTTAGTGATATATAGTTAGTATACAGTG
ATGATTATAGGTGAGGTGATGATGATTGTTTAATGTTGTATAGTTAGTGTAAGTTCAATA
TTATAATCTTATTGTATGTAAGGTATGGGTTGTTTGTGTTGAATGTTGGTTGGAATTGAG
GTCTGGATTTTCACGGAAGTACTGnnnnnnnnnnCTGCCCGGGCG
>cl5ct23cn26 COVERAGE:1.0; CRAWID: 1; TOTAL_ESTS:1; ESTS:vale49365; LENGTH:
405bp; MAP: ; ALT_CONSENSI:0
TTTTGAGTTCATACATACTGCCCACACTTATTAAAAACAAAAAAAAAACACATTGTTCCA
CGAGCCCACACAACATTCCTCACATTCCATGCGGCATCCGGCCTGCTCCTCCACTCCTCC
TCTCGTGCAGTCCGTGGGACGCACGGCAATGAGGCCGGCACCGCGTGTGGACAAAAAAAG
CAAACAAAAGAGGGAGAAGCACGTCACGCATGACGGTTTGCAGCAGGCGACCGTTTTTTT
GGGGGACAACACACAAACAGGGGTGCTGATTATTTCAGCAGAGCACCAGCAAGAAAAAAC
ACCAAAAAACAAAAAACAGGAGAAATGCAGAAAGCAATAAATCAACAGAAAAAACAAAAA
ACAGAATAAAAAAGAAAAAAACAGAAAAAAAGAAAAGAAAAAATG
>cl5ct24cn27 COVERAGE:0.99; CRAWID: 1; TOTAL_ESTS:2; ESTS:
vale49532,vale49493; LENGTH:421bp; MAP: ; ALT_CONSENSI:0
CAACATCCCTCACATTCCATGCGGCATTTTTCCTGCTCCTCCACTCCTCCCTTCGGGCAG
TCCGTGGGACGCACAGCAATGAGGCCGGCACCGCGTGTGGGCAAAAAAAGCAAACAAAAG
AGGGAGAAGCACGTCACGCATGACGGTTTGCAGCAGGCGACCGTTTTTTGGGGAAAACAC
ACAAACAGGGGTGCTGATTATTTCAGCAGAGCACCAGCAAGAAAAAACACCAGAAACAAA
AAACAGGAGAGATGCAGAAAACAATAAAAAACAGGAAAAAAACAGGAAAAAACAAAAAAC
AGAAAAAAAAGAAAAAGAAAAAACAGAAACGTAAAAAAGAAAAAAAGAAAAAAGAAAAAG
GAGAAAAAACGTTGAAAAGAGAAAGTATCCTTGACAAAAACTCATTCAGTATTGCTGGGA
C
>cl5ct25cn28 COVERAGE:0.99; CRAWID: 1; TOTAL_ESTS:2; ESTS:
vale49228,vale49253; LENGTH:896bp; MAP: ; ALT_CONSENSI:1
GTGTGAGCACCTCTGCTCATTCGGGCAGTTTCGGGATGGGTGTCCGTGAAAGCACCTCTC
TGCAGGAAGAAGTGCCACCACCTCTCGGGACTGAAGATATTCCGAAGGCCGACGTGGAGC
GACCGATCCATGAAGAAGAAGCGACCTCTCCGGAAGGGGCCACTGAGTGGCAGACGCAAG
AAACCACTGCGCCTTTGGTTGAAAATGAAGACAGCGAGGACGTTAGCACCGCATCGGGCA
ATACAAGCACCCTGCCTGGGGAAACCGAGATCTCATCGAAGTCCAACGCAACAGCACCCT
CGGACGCTGGCATTTTGCTTGAGAACGGGCATTTCGAGGAGTTGGCGGGCATGGCTCTAT
TTGCTGAGAGCACCGCGCATGGGTGTGTGTCTCGGGTGCTGTTGCTGATGCTTCTGGGGC
TGTGGGGCACTGCGGCTCTCTGTTGAATGAATTGGGGAGAGAAAAATTGCAGAATTCTGT
GGAGCAACAACTGAGCGGCATCCCAGCAATACTCAATGAGTTTTGCCAATGATACTTTCT
CTTTTCTACGTTTTTTCTCTTTTTTCTTTTTTTCTTTCCTTTTCTGTGTTTTTTCTTTTT
CTTTTTTTTCCTTTTTTTTTGTTTTTGGTGTTTTTTATTGCTGGTGCTCTGCTGAAATAAT
CAGCACCCCTGTTTGTGTGTTTTCCCCAAAAAACGGTCGCCTGCTGCAAACCGTCATGCG
TGACGTGTTTCCCCCTCTTTTGTTTGCTTTTTTGCCCACACGCGGTGCCGGCCTCATTGC
TGTGCGTCCCACGGACTGCACGAAGGGAGGAGTGGAGGAGCAGGCAAGATGCCGCATGGA
ATGTGAGGGATGTTGTAAAGGATGGTGGGAGAGTACCTGCCCGAAGGCCGCTCGAA
>cl5ct26cn30 COVERAGE:0.96; CRAWID: 1; TOTAL_ESTS:4; ESTS:
vale49164,vale49252,vale49563,vale49335; LENGTH:628bp; MAP: ; ALT_CONSENSI:
0
TTTGGAGGGCGTGCACGACGAGCCTTTTATATGCCAACACGCTCGCCAGCGAAGAACAAT
ACGCTGACCGTGACGAAGACACGCATGCCCACACTGCCGTGGGCACGAACAGCGGCCCCG
ATTCATTCAGCAGCACTAACGTGTCGGGTGGCGCTGATGCCGCGCCGACTCCTTCGAGCG
CAGCGCCTGGGGAAACCAAGATCCCATCGGAGCTCAACGCAACAATACCCTCGGACCACG
ACATTTTGCTTGAGTTCCGCGATTTGGCGGCCATGGCTCTAATTGGTGACAGCACCGTGC

ATGCGTGTGTGTCTCGGGTGTTGTTGTTGCTGCTTCTGGGGCTGTGGGGCACTGCGGCTC
TTTGCTGAGGTGTGAGGGAGGGTGGAATTGCGGCTTTTCGTGGAACGACAACTGTGCAGC
TTCCCAGCACTACGCAATCAAATGTCAAGAATACCTCTTTTTCTTTTTTTTTTACTTTTT
TTTCTTTTTTTACTTTTTTTTGTTTCATTTCTTTTTTTTCCTTTTTTCACTTTCTTTTGG
TTACATAATTTTTTTTTTTGCTGGGGCTCAGTTGAAATAATCAGCACCCCTGTGTGGTTT
CCCCCCCAAATGGGCGCTTTCTGCACAT
>cl5ct27cn31 COVERAGE:0.96; CRAWID: 1; TOTAL_ESTS:6; ESTS:
vale49230,vale49553,vale49280,vale49316,vale49318,vale49323; LENGTH:553bp;
MAP: ; ALT_CONSENSI:0
TAGCGTGGTCGCGGCCGCGGGGTCGCGGCCGTCCTGCAGATTTCATGCTTACATTTCCTG
ATATCATTGTCCCCTCCACCTCAGCTGGTGTGGGAAAGGTGGAAGAAGAGGCACCCGACA
GCGGTGCCTTGGCGTCTGCATCGTCACAAACGCAGAACGCGGGCAGTCATGAGTTAATTG
GAACCGAGATGCCTTTCAGTGGGGAGCACTTTCCTCCCAATATAGATTTACCGCTGATGG
GGCAGGTGGACACGGCAGATGAAGAATCTCCGCGGATCGGCAACACCGATGATCAGGCGC
CGCACAGCGTTTCACCTGATGTTTCGGAGAGCGTGGGCACGAACAGCGACCCCGATTCAT
TCAGCAGCACTAACGTGTCGGGTGGCGCTGATGCCGCACCGGCTCCTCCGAGCACAGCGC
CTGGGGAAACCAAGATTCCATCGGAGCTCAACGCAACAATACCCTCGGACCACGACATTT
TGCTTGAGTTCCGCGAGTTGGCGGCCATGGCTCTAATTGGTGACAGCACCGTGCATGTGT
GTGTGTCTCGGGT
>cl5ct28cn32 COVERAGE:0.94; CRAWID: 1; TOTAL_ESTS:6; ESTS:
vale49168,vale49274,vale49291,vale49281,vale49204,vale49509; LENGTH:596bp;
MAP: ; ALT_CONSENSI:0
AAAAAAACCAAAAGAAAAAACAATTGACTCGGCAGATATGTCATATCATGTGCCACGCAG
CACAATGCAACCGTGCTTTTGAGATTGTACATACTGCCCGCACACACAAAAATAAAAAGG
AATTGTACTCTCCCACCAGCCCACACAACATCCCTCACATTCCATGCGGCATCCGGCCTG
CTCCTCCACTCCTCCTTTCGTGCAGTCCGTGGGACGCACAGCAACGAGGCCGGCACCGCG
TGTGGGCAAAAAAAAGCAAACAAAAGAGGGGGAAGCACGTCACTCGTGACGGTTTTTAGA
AAGCGACCATTTGGGGGAAAACACACAAATAGGAATGCTGATTATTTCAACAGAGCACCA
ACAAAAAAAAAAAAAAGAGAAAGGAGAACAGGAAAAAAAAGGAAAAAAACAAAAGAAAGT
AAAAAAAAAAAAAAACAGGCCTAAAAGAAGAAGAAAAAGAAAGTCTGCTTGACAAAGTTG
AGTGAGTGATGCTGGGAGCTGCACAGTTGTTGTTCCACGAAAAGCCGCTATTCCACTCCT
CCCTCAGACCTCAGCAGAGAGCCGCAGAACCACACACCTCGGCCGCGACCACAATA
>cl5ct29cn33 COVERAGE:0.95; CRAWID: 1; TOTAL_ESTS:14; ESTS:
vale49163,vale49156,vale49546,vale49548,vale49549,vale49128,vale49125,vale4
9260,vale49184,vale49561,vale49302,vale49331,vale49593,vale49574; LENGTH:
864bp; MAP: ; ALT_CONSENSI:0
GGAAGAAGCGCCACCACCTCTGGGGACTGAAGACATCCCGAAGGCTGACGTGGAGCGACT
GATCCACGAAGAAGAAGCGACCTCTCCGGAAGAGGCCACTGAGCGGCAGACGCAAGAAAC
CACTGCGCCTTTGGTTCAAAATGGAGACGGTGAGGACGTTGGCACCGCACCGGGTAATGC
AAGCACCCTGCCTGGGGAAACCGAGATCTCATCGGAGTCCAACGCAACATCGCTCTCGGA
CCATGGCATTTTGCCTGAGCACGAGCATTTGAGCGACTTGTCGGGCATGGCTCTATTTGC
TGAGAGCACCGTGCATGGGTGTGTGTCTCGGGTGTTTTTGCTTCTACTTCTGGGGCTGTG
GGGCACTGCGGCTCTTTGCTGAATAAATTTGGGGAGAAAAATTGCAGAATTCTGTGGAGC
AAAAACTGAGCGGCGTCCCAGCAATACTCAATGAGTTTTGTCAAGGATACTTTCTCTTTT
TTTATTTTTCTTTTTTTCCTTTTTTCTTTACTTTTTTTTTTCGTTTTGTTTTTTTATTGT
TTTCTGTTTTCTTTTTTTTATGTTTTTCTTTTCTTTTTTTTTTCTTTTTTTGTTTTTCTTT
TTTTTCTGTTGTTTTTTTTTTCTTGCTGGTGCTCTGTTGAAATAATCAGCACCCCTGTTTG
TGTGTGTTTTTCCCCCCAAAAAACGTTCGCTTGCTAAAAACCGTCACGAGTGACGTGCTT
CTCCCTCTTTTGTTTGCTTTTTTTGTCCACACGCGGTGCCGGTCTCATTGCTGTGCGTCC
CACGGACTGCACAAAAGGAGGAGTGGAGGAGCAGGCCGGATGCAGCATGGAATGTGAGGA

ATGTTGTGTGGGCTAGTGAAAGAA
>cl5ct30cn34 COVERAGE:0.96; CRAWID: 1; TOTAL_ESTS:13; ESTS:
vale49551,vale49554,vale49263,vale49257,vale49187,vale49557,vale49295,vale4
9284,vale49507,vale49352,vale49383,vale49522,vale49528; LENGTH:999bp; MAP:
; ALT_CONSENSI:1
TATTTCGGCCGGCCCCCGGCCCAGGTACTTCACTTGTATGTTTTGGAGGGCGTGCACGAC
GAGCCTTCCAATGCCAACACGCTCGCCAGCGAAGAACAATACGCTGACCGTGACGAGGAC
ACGCATGTTTCGGAGAGCGTGGGCACGAACAGCGACCCCGATTCATTCAGCAGCACTAAC
GTGTCGGGTGGCGCTGATGCCGCACCGGCTCCTCCGAGCACAGCGCCTGGGGAAACCAAG
ATTCCATCGGAGCTCAACGCAACAATACCCTCGGACCACGACATTTTGCTTGAGTTCCGC
GAGTTGGCGGCCATGGCTCTAATTGGTGACAGCACCGTGCATGTGTGTGTGTCTCGGGTG
TTGTTGTTGCTTCTGGGGCTGTGGGGCACTGCGGCTCTCTGCTGAGGTGTGAGGGAGGGT
GGAATTGCGGCTTTTCGTGGAACAACAAATGTGCAGCTTCCCAGCACTACGCACTCAAAT
TTGTCAAGAATACTTCTTTTTTTTTCTTTTTTTCTTTTTTCATTTCATTTTTTTTTCTTT
TGTTTTTTTTACTTTTTCATTTCATTTCTTTTTTTTTTTTTTTTTTTGCTGGTGCTCTGT
TGAAATAACCAGCACCCCTGTGTGTTTTCCCCCAAATGGTCGCTTGCTGCGCACCGTCAT
GAGCGATGTGTTTCCCCCTCTTGTTTGCTTTTGCTTTTGTTACCCACACGCGGTGCCGGC
CTCATTGCCGTGCGTCCCACGGACTGCACGAGAGGAGGAGTGGAGGAGCAGGCCGGATGC
CGCATGGAATGTGAGGAATGTTGTGTGGGCTCGTGGAACAATGTGTTTTTTTTTGTTTTT
TCTGTGTGGACAGTATGTATGAACTCAAAAACACGGTCGTATTGTACCTCGGCGGAGACC
ACGTTAAATTCCCGCCAAATCGAAAATTTTTTTCTTTTTTTTCTTTTTTATGTTTTAAAT
AAATGGTAAAAAAAAACCTCGGCCGCGACCACGCTAATA
>cl6ct31cn36 COVERAGE:0.96; CRAWID: 1; TOTAL_ESTS:5; ESTS:
vale49205,vale49315,vale49330,vale49583,vale49375; LENGTH:396bp; MAP: ;
ALT_CONSENSI:0
TATATTTATTGTCTCACAAAGTCGAGCGACTCTACGACGAATGATGATGATGGTGACTGT
GCGGCGCCGCGTGGTGTGCGACCTGCTGATCCTCGCGCTCTTGTGCTGCTGCTGCTGCTT
GTCCGTCTGCGGGACTGCTGTGCCGGCAAAAACCTTTGTGTTTGTGCATTTTTCGTGTCC
CGGGACTGACGGTAAGTTGAGTTGGCATCTTCGAGGCGAGGAGGAATGGAAAAAGTGCCC
TAAGAAGCCTGGAGAAGTCGATCAAGACAGTGATGAGCGTGCACGGCTGTGCGTTGCCGG
TGGGGACTTCTACAGAGCTTGGAAAAGTGGAAGGAGAGTATTTTGCGCTCCTCCCAGTGA
CCAAGTAGAGTTTATGTTCGAGATAAAATTTGCGAC
>cl7ct32cn37 COVERAGE:0.99; CRAWID: 1; TOTAL_ESTS:2; ESTS:
vale49159,vale49141; LENGTH:290bp; MAP: ; ALT_CONSENSI:0
ATTATTTTCGGGATTTTGGTAAATTTCACnnnnnnnnnnACAAGATTGGTATAGATTTAT
TATAACGGTGTATTTGATATGTGTATATGACGGTGTGGAGATAGACTATAAATGTGTTCT
TGAAAGTTATATAGAGACTTGTTGAAATTTATTGGATATGAGGATAATGGTATGTTGTCT
ATAATTGGGTATGGGTGGTTATGGCGGGGGTTGCGTTATAAGGTTGTGTTACAGGGAGAA
TGTGGTCCGAAAAATTCCAGGAAAGTTGGnnnnnnnnnnCTGCCCGGGCG
>cl8ct33cn38 COVERAGE:0.96; CRAWID: 1; TOTAL_ESTS:6; ESTS:
vale49121,vale49188,vale49243,vale49322,vale49602,vale49617; LENGTH:418bp;
MAP: ; ALT_CONSENSI:0
TAGCGTGGTCGCGGCCGATGTACACCATTTTGGGCTGAAGAGGTGTGTGCGGGCTGTGCG
TGCCAGCACTCAGCCACGGGGACATGTGCACGTCTACCGTGGTCCGATGTATAAAGTAAG
AGTAATTATGATGATTGTGTTTTGTTTGCTTGCTTCTGCTGCCATGCTGTGTGCTCCGCG
TTGTGCACCACATCCGTCACGTTGAACGCATCCCAGACACACCCATATGCATGCGCACTG
GAGCCTTGGGTTTGTTTTTTCTTGTTCTTTAATTCTTTTCTTAATTCTTTTTTTAATTCT
TTTTTTCGTTGTTTCTGCTTTTATAGCTTCTGTGTTGTGGGGCGGTGTGCGAGTTCTGCA
ATTGTACCTGCCCGGGAAAAAAAAAAAAAAAAGCTTGTACCTCGGCCGCGACCACCCTA
>cl9ct34cn39 COVERAGE:1.0; CRAWID: 1; TOTAL_ESTS:1; ESTS:vale49235; LENGTH:

477bp; MAP: ; ALT_CONSENSI:0
ACAACATCCCTCACTTTTATTGCGGCATCCTTTCTTTTCTTCCAATCCTCCCTTCGTGCA
GTCCGTGGGACGCACAGCCGCAGGGCCGACATCGCGTGTGGGCAAAAAAAGAAAAAAACA
ATAGGAAAAACACATCACTCATGACAGTGTGCAGCAACCAAAAATTAAAAAAAAGAAAAA
AGCATTACTGAGTATTACAACCAAACACTAAAAAAAAAAGAAAAAGAAAAGAACCCGCGG
GGAATGCGCCACACAATCTCCGTGAGGAGTCGCAATGTCACACTCTCCGGCTCACAAGAC
TGCCACAGGTGCACACAGTCCCAGAAGAAGCAGCAACACACAAGACACGCGCACACGCGC
AGTGCTATCACCACTCAAATCATGCAGGATCAATTTCGCGTCGGTGATCAGGGCGAGGCG
GTCCTCTGGACTCATACTGCCATCGGATGCTGAGTGATTGGTTTCTGGAACGGAAGT
>cl9ct35cn40 COVERAGE:1.0; CRAWID: 1; TOTAL_ESTS:1; ESTS:vale49595; LENGTH:
483bp; MAP: ; ALT_CONSENSI:0
TAGCGTGGTCGCGGCCGAGGTCACACTGTCTCCATAAGTGACACTTCCGTTCCAGAAACC
AATCACTCGGCATCCGATGGCTGTATGGGTCCAGAGGACCGCCTCGCTCTGATCACCGAC
GCGAAATTGATCCTGCATGATTTACGTGGTGACAGCACTGCGCGTGTGCGCGTGTCTTGT
GTGTTGCTGCTTCTTCTGGGACTGTGTGCACTTGTGGCAGTCTTGTGAGCCGAAGAGTGT
GGAATTGCGACTCCTCACGGAGATTGTGTGGCGCATTCCCCGCGGGTTCTTTTTTTTTCC
TTTTTTTTTGTGTGTGCGTGTTTTTTTTGCAATACTCAGTAATTCGTTTTTTTCTTTTTT
TTAATTTTTGGTTGCTGCACACTGTTATGAGTGATTTGTTTCCCCCTCTTGTTTTGTTGC
CCACACGCGGTGCCGGCCCTGCGGCTGTGCATCCCATGGACTGCACGAAAGGGAGGATTG
GAA
>cl9ct36cn41 COVERAGE:0.99; CRAWID: 1; TOTAL_ESTS:2; ESTS:
vale49603,vale49608; LENGTH:496bp; MAP: ; ALT_CONSENSI:0
TTCCAGCGGCCGTTCCGGGCAGGTACTGTTCCACCTTTCCACACAACACTCTTCATTTTT
ATTGCGGCATCCTGCCTTTTCTTCCAATCCTCCCTTCATGCAGGCCGTGGGACGCACAGC
CGCAGGGCCGGCACCGCGTGTGGGCGACAAAAGCAAACAAGAGGGGGAACACGTCACTCA
TGACGATGTGCAGCAACCAAAAGTTAAAAAAAAAAAGAAAAGAAAAGAATTACTGAGTAT
TACAACAAAACAGTAAAAAAAAAAAAAAAAAGGAAGAACCCGCGGGGAATGCGCCACACA
ATCTCCGTGAGGAGTCGCAATTTCACATTCTTCGGCTCACAAGACTGCCACAAGTGCACA
CAGTCCCAGAAGAAGCAGCAACGCCCGGGAGACGCACGCAAACGCAGTGCTGTCACCACG
TAAATCATGCAGGATCAATTTCGCGTCGGTGATCAGGGCGAGGCGGTCCTCTGAACTCAT
TCTGCCATCGGATGCT
>cl9ct37cn42 COVERAGE:0.99; CRAWID: 1; TOTAL_ESTS:4; ESTS:
vale49133,vale49193,vale49560,vale49304; LENGTH:836bp; MAP: ; ALT_CONSENSI:
0
CCACACAACATCCCTCACATTTCATTGCTTTATTCGGCCTTTTCTTCCAATCCTCCCTTC
ATGCAGTCCGTGGGACGCACAGCCGCAGGGCCAGCACCGCGTGTGGGCAACAAAAAGCAA
ACAAGAGGGGGAACACGTCACTCATGACAGTGTGCAGCAACCAAACATTAAAAAAAAAAA
AAAGAATTACTGAGTATTACAAAAAAACACTAAAAAAAAAAAAAAAAGAAAAAAAAAGGAA
GAACCCGCGAGGAATGCGCCACACAATCTCCGTGAGGAGTCGCAATTTCACATTCTTCGG
CTCACAAGACTGCCACAAGTGCACACAGTCCCAGAAGAAGCAGCAACGCCCGGGAGACGC
ACGCAAACGCAGTGCTGTCACCACGTAAATCATGCAGGATCAATTTCGCGTCGGTGATCA
GGGCGAGGCGGTCCTCTGGACCCATACTGCCATCGGATGCTGAGTGATTGGTTTCTGGAA
TGGAAGTGTCATTTTTGGCGGCAGTGCGTGGACTCAATTCTTCCGTAACATTCTCAGCAC
TCGATTCACTGCCCGCAAGAACACGGACAGACTGAGAGTCAGTCGGCTGTGCAATTGGAG
CCTCTTCAACGTCTGCCAAGGAAGCCGTCAGCTGTGTGGCCGTCTGCAATGCAGCTGCCG
GCTGTGCAACGTCCAGCTGTCCGACCTTCTGCTTTTCTTCCTCCGTCGTTGTGACTTTCA
CCGCAGCGTGCAGTTCTTCGGCAGCCGGATGTCGAATAGGGACTGTCCTGGCGAAGAGGT
CTCCGAGGTCATCATCATACAGTAAACAATTGTACCTGCCCGGGCGGCCGATCGAA
>cl9ct38cn43 COVERAGE:0.96; CRAWID: 1; TOTAL_ESTS:6; ESTS:
vale49256,vale49308,vale49581,vale49499,vale49505,vale49619; LENGTH:824bp;

MAP: ; ALT_CONSENSI:1
TAGCGTGTTCGTTGCCGAGGTACAACCGCGCTCTGTTTGGCGATGACCTCGGAAACCTCA
ACGCCTGGTTTGTTCCTATTCGACATCCGGCTGCCGAAGAACTGCACACTGCGGTGAAAG
TCACAACGACGGGGGAAGAAAAGCAGACGGCCACACAGCTGACGACTTCCTTGGCGGACG
TTGAAGAGGCTCCAATTGCACAGCCGACTGACTCTCAGTCTGTCCGTGTTCTTGCGGGCA
GTGAATCGAGTGCTGAGAATGTTACGGACGAATTGAGTCCACACACTGCCGCCAAAAATG
ACACTTTCGTTCCAGAAACCAATCACTCAGCATCCGATGGCAGTATGAGTCCAGAGGACC
GCCTCGCCCTGATCACCGACGCGAAATTGATCCTGCATGATTTACGTGGTGACAGCACTG
CGCGTGTGTGCGTCTCCCGGGCGTTGCTGCTTCTTCTGGGACTGTGCGCACTTGTGGCAT
TCTTCTGAGCCAAATAGTGTAGAATTGCGACTCCTCACGGAGATTGTGTGCTGCGTTCCC
CGCGGGTTCTTCTTTTTTTTTCTTTTTTTTCTTTTTTTTTCTCTTTTTTTTCTTTTTTTC
TTTTTTTCTTTTTCTTTCGTTTTCAGTGTTTCGTTGTAATACTCAGCAATTCTTTTTTTC
TTTTTTTTTTAATTTTTGGTTGCTGCACACTGTTATGAGTGATTTGTTTCCCCCTCTTGT
TTTGTTGCCCACACGCGGTGCCGGCCCTGCGGCTGTGCATCCCATGGACTGCACGAAAGG
GAGGATTGGAAGAAAAGGCCGGATGCCGCAATGTAAGTGAGGGA
>cl10ct39cn45 COVERAGE:0.99; CRAWID: 1; TOTAL_ESTS:2; ESTS:
vale49550,vale49293; LENGTH:463bp; MAP: ; ALT_CONSENSI:0
AGAAGTATGATTTAACAGAAGAGGCATACAGCAAACGGGAGGACAATGCTCGGGCTTTCC
GCCAACTCATGATGGCAAGACAACGTGAGGAGGCCGAAAAGGCTGGTAATCCTCTCCCAA
AGGAACTGGACGCAGACAGCTACAAGGAAACGGCTGAGAAGATCCACGTCGATGATCGTT
GCCAGTGCCAGCCTGGAGACCGTCTGGGAACGGTGCGGCTTGTTGGCCGCGTTGCGTCGC
TGAAGCCAGGCTATTGGATTGGCGTTGAGTTTGACGAGCCTGTGGGGAAGGGTGACGGTA
GTGTCAAGGGGACGCGTGTGTTCGAATGTCAGCCGAAATATGGCGGGTTTTTACGTCCGG
ACCAGGTGACAGTCGGGGATTTTCCACCAGAGGAGGTTTAGTGAAATGCGGTCCATAATT
TTGAATCATTTTACCGAAGATAAAACAAGACGCTTTGCGCATA
>cl11ct40cn46 COVERAGE:0.98; CRAWID: 1; TOTAL_ESTS:2; ESTS:
vale49248,vale49319; LENGTH:337bp; MAP: ; ALT_CONSENSI:0
ACAGCCGAGGACGGCCTTGCCCCAAGTTCGAGCGAAAACATCACCGTCTAGGAGGAGTCG
CCTAAGCAAACAGCCGACGGCGGCTCTTATCCAATGCTAAGCGAAAACGTCACCGTCAAG
GAGAAGTTGCCTGACCAAACAGCTGAGGACGGCTCTGCCTCAAGGCCGAGCGAAAACTTC
GCCGTCAGCCCGCTGACATCCGCAGAAGGGGCAGCCAATGAAACTTCCCACAACAGTAAT
GAGGGTGACAGCCAATCCGACGCCAGTGGCGAAGGCAACAGCACCGTTACCGGCACGAAT
CCGCTCCGACCGACCAGAGACGAAAGTGCAAACGAAA
>cl11ct41cn47 COVERAGE:0.99; CRAWID: 1; TOTAL_ESTS:2; ESTS:
vale49279,vale49196; LENGTH:469bp; MAP: ; ALT_CONSENSI:0
TACCAGCGGCCGCCCCGACGGGCAGCAAACTGCTTAAGGCGGATCTTATCCAATGCTAAG
CGAAAACGTTTCCGTCAAGGAGAAGTTGCCTGACCAAACAGCTGAGGACGGATCTGCCCA
AAGGCCGAGCGAAAACATCGCCGCCAGCCCGCTGACATCGACAGACGCGAAAGGGCACGT
GGCATCGGCACCCGAAGCTTCCGGTCGGCCGTCCTCTTCTCCATCCGGCGACGGCGCAGC
CAATGAAACTTCCCACAACAGTAATGAGGGTGACAGCGAATCCGACGCCAGTGGCGAAGG
CAACAGCACCGTTGCCGGCACGAATCCGCTCCGACCGACCAGAGACGAAAGTGCAAACGA
AAGCACAGAAGATATCGCTGCTTTGTTGCAGAATTTTGTCGTTCTGGACGGCAGCGACGT
CACCACTATGTGGGTGCGCACGCCCCTGCTGCTGCTCGTCATCGCCCTT
>cl12ct42cn48 COVERAGE:0.98; CRAWID: 1; TOTAL_ESTS:2; ESTS:
vale49262,vale49197; LENGTH:219bp; MAP: ; ALT_CONSENSI:0
TGGTCGCGGCCGAnnnnnnnnnnGCATGGGTTGCGGTTGTTATTTATGGTGTTATTTGAT
TTATGGTTTTGCGTATTGGGTATTAGTGGCTATGATTATAACAACATTATTATAGAGATA
GGTGGTAGAGTGTTCAGTTTGTTTATTGTATGTATATCATGTTTATACTGTTATTATTAT
TTATATTCTTGCGTGGTGGTTGGGGTTGGGTGTGTATAG
>cl13ct43cn49 COVERAGE:0.97; CRAWID: 1; TOTAL_ESTS:4; ESTS:

vale49171,vale49177,vale49604,vale49621; LENGTH:355bp; MAP: ; ALT_CONSENSI:
0
TTCGAGCGGCCGCCAGCGCGGGCCCGGGCAGGTGCATTnnnnnnnnnnCCCAAATTTCAC
GGGAGGTGGGGTTTTATTTTTGTTGGTGTAATATAGGTGCTGTGTGGCTTGGTGAATTAA
GATGATTATTAGATTTGTTTATTTGTGTGATTGGATGGGTGATAGATGGTAGTATACAAA
GAGGTGAGGTGTTTATTCATGTGTGAGGGGTTATTGCATGTTTGTTATGGTAATTTATTA
ATGTTATATGTTATTTTGTTATTCGGTGGTGTGGTGGTAATGGGTATGGTGTGTTTCGTA
GTAAAATTGGGGTCTGGATTTTCACGGAAGTATGGnnnnnnnnnnCTGCCCGGGC
>cl14ct44cn50 COVERAGE:0.98; CRAWID: 1; TOTAL_ESTS:2; ESTS:
vale49287,vale49346; LENGTH:373bp; MAP: ; ALT_CONSENSI:0
AAAACCAAAATTAATTGAAGAATGGGGAAATAATAAAAAAAAAAAAAAAAAAGGATTTTTA
CTAAAGAATGGGAAACCAATAATAAAAAAGGATTTTTACTATCGGGTGCTGCGCGCCAAC
CCCATCGGACGACACCTGCGATGGAGAGCGCGGTTTTCGCAGTGTCGTCAGCAGCAACGC
AGTCCTGCACCCTCGTTTTGCGTTTTATTTTGCCGTAAGATATATTGGTCTCACGTCTCA
CACGGTGCCACACGACACACTCACGCGGCTTGACGGTTGCCAGGGGTGGCAACCTCAATC
GGCGACTCCCGTTCTGGAAGCGCGGACTCTTCGTTGGCGTCTCCGTGGTTCTGAGGAGTG
CTCTCATAACACG
>cl14ct45cn51 COVERAGE:0.97; CRAWID: 1; TOTAL_ESTS:4; ESTS:
vale49180,vale49286,vale49210,vale49239; LENGTH:406bp; MAP: ; ALT_CONSENSI:
0
TAGAGCGGGCGTCCCGGCCGGGACCAAACCATAATTTATAAGAGAATGGGGAAACAATTA
AAAAAAAAAAGGATTTTTACTAAAGAATGGGAAAACAATAATAATAAAAAAAGATTTTTA
CTATCGGGTGCTGCGCGCCAACCCCATCGGACGACACCTGCGGTGGAAGGCGCGTTTTTC
GCAGTGTCGTCAGCAGCAACGCAGTCCTGCACCCTCGTTTTGCGTTTTATTTTGCCGTAA
GATATATTGGTCTCACGTCTCACACGGTGCCACACGACACACTCACGCGGCTTGACGGTT
GCCAGGGGTGGCAACCTCAATCGGCGACTGCCGTTCTGGAAGCGCGGACTCTTCGTTGGT
GTCTCCGTGGTTCTGAGGAGTGCTCACCTCGGCCGCGACCACGCTA
>cl15ct46cn52 COVERAGE:0.99; CRAWID: 1; TOTAL_ESTS:2; ESTS:
vale49296,vale49251; LENGTH:507bp; MAP: ; ALT_CONSENSI:0
ACATACGCTCCGCGGACGGTGTCACATTGCGTGGATTTATTTTGTGGCCATGGCCTGGAC
AACACGCAGCACAGCAGGGAGCCACAACCGGTGCTTTTAGGAGCCAAGGTTCCGGTATGA
ACGTCGGGATTCCCTTGTCGTCTAGCTCGACGACGCTTAGAGACTCACTGTTCCCATCGC
CGCTTTACGCCGTCATATATTTTCACGGAAACTCGGGGAATGCTGGCCACCGCATACCAA
TTGCCGAGCTTCTCACCTCGAAAAATCCCTGCGCAGTTCTTATGGTGGATTACCGCGGAT
TTGGTCTTAGCGATGCTGTTCCGCCAACGGAGGAGGGACTCAAGTTGGATGCGCAGGCAT
GTTTGGAATACCTCTGGAACCATCCTCGAATACCACAGGGGCGAATCTTTGTCATGGGGA
CAAGTCTGGGTGGTGCTGTCGCCATTGATTTGGCGTCGCGGCGAATGAACATGAAACGTA
TCGCCGGGGTGATTATTGAAAACACCT
>cl16ct47cn53 COVERAGE:1.0; CRAWID: 1; TOTAL_ESTS:3; ESTS:
vale49297,vale49300,vale49605; LENGTH:354bp; MAP: ; ALT_CONSENSI:0
TAGCGTGGTCGCGGCTTAGGTACATTATTTCGTGGATTTTGCCAAATTTCACGGGAGGTG
GGGTTCTTTTTTTGTTGGTGTAATATAGAGCCTGGGTTGCGGTAGGGTGATAGTAGTGTT
GGTATATTGTATAATGATTCTATAGTTATTGATTCATAATGATGGTAATTATAACTATGA
CATAGAGGATAGAATATGTTTGTGTGAATGGATAGTTAATGTTCTTCGATAATGTTATTG
TATTGTTATCATTATAGTTGTGTTGGTAAGATGGTGTATGGTGTGCATGTGGTTACTCAG
GGGTAAGTTTGTGGTCGGAAAAATTCCAGGAAATTCTGnnnnnnnnnnCTGCCC
>cl17ct48cn54 COVERAGE:0.98; CRAWID: 1; TOTAL_ESTS:2; ESTS:
vale49313,vale49312; LENGTH:380bp; MAP: ; ALT_CONSENSI:0
ACTGCTATGCGATGAGGCTTGGGACATCTACGACGATCCGGTCATTATTGAAATGCGCAT
TTTTGACAGTGATCCGAATAATGCTGCGTTCCAGATGAAATTTGAGACGCACGGGGATCT

AAAGAACTGCAGTTTGGGGGAGGCACCGAACATTGATAGGGTGACCGCACAGGGTGTTCG
CCAGGCAGAGAGCGGCGTCGAAGGTTCGCCGGGGGACTCAGCTGAGGCACCAAGGAATAA
ATTGCCTGACCCAACAGCCAAACGCCGCCCTGATCCAACGACGAGCGAAACCGTCACCGC
CAGCCCGCTGACATCCACAGAAGGGGCAATTCAAAGCCGGCAACGTGAAGACGAGAGTGT
CGTGACTAAAGGAGTTCACG
>cl18ct49cn55 COVERAGE:0.99; CRAWID: 1; TOTAL_ESTS:2; ESTS:
vale49227,vale49390; LENGTH:403bp; MAP: ; ALT_CONSENSI:0
ATAGATAGATTGTATACTTAGTTAATGTATGTTACAGAATTGTTTATAATGTTATGAGGG
GTTAGTCTGTATGAGGGATTAGTCTGTATGAGGGATATGTTTGAGTGTTGGGTATTGTAT
TCTATGTTAAAAGGGTGGTCTGAAATTTGCGGGAATTCTGGnnnnnnnnnnTAGCGTGGT
CTCGGCCGAnnnnnnnnnnCTGGCTGGTGGGGTTAGAGGATGGGATTGATTTGATAGATT
TGTATGTTGCAGTTATTGATGTGGGTTGATCGTAATGGAAGGTGATAGTGATGGTGAGAT
TTAAATTGGATGTGTTTGCTTTGATATTAGATTGTTTTGTTAACGTTTTAATTTGAGGTG
TGGTAGGGTATATGTGATTGGTGTGGCGGACGTGTGGTATAAA
>cl19ct50cn56 COVERAGE:0.98; CRAWID: 1; TOTAL_ESTS:3; ESTS:
vale49354,vale49381,vale49572; LENGTH:846bp; MAP: ; ALT_CONSENSI:0
TATTTCCAGCGGCCGCCCGGGCAGGTACCCGGTGCGTGTGTGACGGCAGTGCAGTGAACA
CAAACAGAAGGTTATTGACACGCGGTGCGAGTGGAGAAGACACGAACAACATGCACAGAG
AGAAAATAAAAACGCATCGTCCTCTCCTCCCCTAGCGCCTCTGCGTGCGTCATCCATCCA
CCACGACAACAGTCGATGCACCTCGGGCCGTTTTTTTTTTTGTTGTGCTTGTTCACACCA
CTGCCTCACGCTTTTTATTACGTCCAATACACACAAACACACAGTAAACAATATGTATAT
GTATGTATTTACTGCACCTCCTCACGGCGTGCACGTTTCTTCTTTGGCACTTCCTGCTTA
ACCTCGTCATCACCATTGCCTCCTGCTGCGGCCTGGGCTTCGCGTTGCCTCTTGGAGCGC
TTGCGGAGGATTTTCTGGTATTGCTCGAGTGCCTCGGCCATGGCGACCATGTTTGTTGGC
GGCCTGTTGCCCGTGTCGAAGAAGTTCAACCGCGCCTCCACCTGCTCCCGCAGCTTCTCA
CCAAAGACAGCCGGAGGAGTTTCCATGAAGCAGTCAATGCGGCAGGCAAGGGCAGCCTTG
TTGGCGAGATAGCGTGAGATCTTCCCGCGGTGCTCCTTCGCTGCGCGCTGAATAAATGTC
GAGTGAAAGATAAGCCCGTATTTCGGTGTGTTGCCCTTACGTTTCTTAAGGGCGCGAAAA
AGTGCCTTCTCGGCGCCAAGGATCTGAATGGTGCTGGCAGGCGCCTAAGCGAGGTTTGTC
AGTGAGCCCGCCTTTGAAATGAGCTTAGCACCGATGTTTTGTCCCACCACGGCCGCGACC
ACGCTA
>cl20ct51cn57 COVERAGE:0.98; CRAWID: 1; TOTAL_ESTS:4; ESTS:
vale49358,vale49355,vale49586,vale49575; LENGTH:544bp; MAP: ; ALT_CONSENSI:
0
TAGCGTGGTCGCGGTTCGGGGTCTACGACGAATGATGTTGGTGACTGTGCGGCGCCGCGT
GGCGTGCGACCTGCTGGTCCTTGCGCTCTTGTGCTGCTGCTGCTTGTCCGTCTGCAGGGC
TACTGCTTCGGAGGTGAATTCTACCATTGCGCCGGCAAAACCATTGGCTGATGTGGTAGT
GTTTTGTCCGGGGACTGACGGTAAGTTGCGTTGGCGATTTCAAGGCGAGAAGGACTGGCG
AAAGTGTGCGAGGAGGCCCGAAGAAGCCCCGGAAGTCAATGGTTACTGCGCACGGCTATG
CAATTTCGCTGGAGACATATACAAGGAACCCGAAATTACCAGACTGTGCAATTCTGCCAG
TAATAAGAATTTTGTTGCCTTCCATATGTCATTTGAGAAGCACGAGGATCTAAAAACCTG
CAGTTTGGTGGAGGCACCCGAAGACTCCAGTCAGTCGTCCTCCGCCTCATCCGGCGACAC
CACCACTGTGTGGGTGCGCACGCCTCTGCTGCCGCTGCTGCTCGTCACCGCCCTTGTCTG
TGCT
>cl21ct52cn58 COVERAGE:0.96; CRAWID: 1; TOTAL_ESTS:2; ESTS:
vale49592,vale49612; LENGTH:130bp; MAP: ; ALT_CONSENSI:0
TGGAAGTGGAAAAAAAAAAAAAAAAGGCCGAAAGGCCATACTTATATCCCCCGGCTGCTAA
GAGGACGGGGAGCAAAAAAAAAAAAAGAAAAAAAAAAAAAAAAGGCTGGAACCTCGGCCGC
GACCACGCAA
>cl22ct53cn59 COVERAGE:0.98; CRAWID: 1; TOTAL_ESTS:2; ESTS:

vale49611,vale49615; LENGTH:380bp; MAP: ; ALT_CONSENSI:0
TAGTGCTTTTCCACAGCACCGTCGCGAATGCACACTCGGAGGAAGGGCTGCTGCAGCCCC
CAGCACTGGGGTATGAGAAACAAAAACAAACAAAAATAAAAACAAAACAAAAGTTCTGGT
AATGGAAAAACGGGCATGCCATCGTCTCCACCGGTGACGAGTTCATATCGTTATCTACCA
GGTGCGCCAGCGACATGGATCGCGACAACGCACGGTTGAACCAAAATAAATCCCTACTTC
TTCTTCGGCGCCTTCTTCACGGCTTTCTTGGGCGCCTTCTTTGCCGCATGCTTCTTGGCA
CTCTTATTAACAGCTTTCTTGGCTGCTGCAGGCTTCTTCTTGGGGGCGGCCCTCTTCTTC
GCAGCAGGCTTCTTCACGGC
>cl23ct54cn60 COVERAGE:0.99; CRAWID: 1; TOTAL_ESTS:2; ESTS:
vale49535,vale49497; LENGTH:353bp; MAP: ; ALT_CONSENSI:0
AGAGCCGTTTGCAACGCCATTACAGCTGTTGTGTCGGCTGAAAAGGCCTGCAGCAACCCA
TCCGAATTGCGCGGTGTTGCGGATAGCAACACTTTCAAGGAGGATGAAAAGAACACCCTT
GGCCTCGAAGCCTCGGTGAATTAAGGGGTGCCAGTTGTATCGGTAGCTCCAAATTATTAT
TGTTTTTCCTTAAGGGAGCTACTCTCTATTATCTGTGTTTGGAACTATGTATACATACAT
GCGTGTGTTTTCACTGTTTTTATCATTATTTTATTTCTTTGCTTTACTGGAGATGAGGAG
CGGGCGATAACGGTCAAATATTAGAAAGTACCTCGGCAGCGACCACGCTAATA

>iib|49153 TcT-E01a10.b1D
>iib|49541 TcT-E01a11.b2D
>iib|49542 TcT-E01a12.b2D
>iib|49543 TcT-E01a13.b2D
>iib|49144 TcT-E01a14.b1D
>iib|49147 TcT-E01a16.b1D
>iib|49150 TcT-E01a17.b1D
>iib|49154 TcT-E01a18.b1D
>iib|49157 TcT-E01a19.b1D
>iib|49160 TcT-E01a20.b1D
>iib|49163 TcT-E01a21.b1D
>iib|49119 TcT-E01a22.b1D
>iib|49120 TcT-E01a23.b1D
>iib|49148 TcT-E01a24.b1D
>iib|49151 TcT-E01a3.b1D
>iib|49155 TcT-E01a5.b1D
>iib|49158 TcT-E01a8.b1D
>iib|49161 TcT-E01a9.b1D
>iib|49200 TcT-E01b1.b1D
>iib|49201 TcT-E01b10.b1D
>iib|49203 TcT-E01b11.b1D
>iib|49205 TcT-E01b12.b1D
>iib|49207 TcT-E01b14.b1D
>iib|49209 TcT-E01b15.b1D
>iib|49211 TcT-E01b16.b1D
>iib|49213 TcT-E01b17.b1D
>iib|49215 TcT-E01b18.b1D
>iib|49216 TcT-E01b19.b1D
>iib|49218 TcT-E01b2.b1D
>iib|49220 TcT-E01b20.b1D
>iib|49222 TcT-E01b22.b1D
>iib|49544 TcT-E01b3.b2D

>iib|49224 TcT-E01b4.b1D
>iib|49226 TcT-E01b6.b1D
>iib|49228 TcT-E01b7.b1D
>iib|49545 TcT-E01b9.b2D
>iib|49164 TcT-E01c1.b1D
>iib|49166 TcT-E01c11.b1D
>iib|49149 TcT-E01c13.b1D
>iib|49152 TcT-E01c14.b1D
>iib|49156 TcT-E01c15.b1D
>iib|49159 TcT-E01c16.b1D
>iib|49162 TcT-E01c17.b1D
>iib|49165 TcT-E01c18.b1D
>iib|49167 TcT-E01c19.b1D
>iib|49121 TcT-E01c2.b1D
>iib|49124 TcT-E01c20.b1D
>iib|49126 TcT-E01c21.b1D
>iib|49139 TcT-E01c23.b1D
>iib|49142 TcT-E01c5.b1D
>iib|49131 TcT-E01c6.b1D
>iib|49136 TcT-E01c9.b1D
>iib|49230 TcT-E01d1.b1D
>iib|49231 TcT-E01d10.b1D
>iib|49234 TcT-E01d13.b1D
>iib|49236 TcT-E01d14.b1D
>iib|49238 TcT-E01d15.b1D
>iib|49240 TcT-E01d16.b1D
>iib|49242 TcT-E01d18.b1D
>iib|49244 TcT-E01d19.b1D
>iib|49546 TcT-E01d22.b2D
>iib|49547 TcT-E01d4.b2D
>iib|49548 TcT-E01d6.b2D
>iib|49549 TcT-E01d7.b2D
>iib|49145 TcT-E01e11.b1D
>iib|49128 TcT-E01e12.b1D
>iib|49143 TcT-E01e14.b1D
>iib|49133 TcT-E01e16.b1D
>iib|49137 TcT-E01e17.b1D
>iib|49122 TcT-E01e18.b1D
>iib|49125 TcT-E01e19.b1D
>iib|49146 TcT-E01e20.b1D
>iib|49140 TcT-E01e21.b1D
>iib|49129 TcT-E01e22.b1D
>iib|49132 TcT-E01e23.b1D
>iib|49134 TcT-E01e5.b1D
>iib|49138 TcT-E01e7.b1D
>iib|49123 TcT-E01e9.b1D
>iib|49550 TcT-E01f13.b2D
>iib|49246 TcT-E01f19.b1D
>iib|49248 TcT-E01f20.b1D
>iib|49250 TcT-E01f22.b1D

>iib|49252 TcT-E01f23.b1D
>iib|49551 TcT-E01f3.b2D
>iib|49552 TcT-E01f5.b2D
>iib|49254 TcT-E01f8.b1D
>iib|49553 TcT-E01f9.b2D
>iib|49168 TcT-E01g1.b1D
>iib|49127 TcT-E01g10.b1D
>iib|49141 TcT-E01g11.b1D
>iib|49130 TcT-E01g12.b1D
>iib|49135 TcT-E01g14.b1D
>iib|49172 TcT-E01g17.b1D
>iib|49176 TcT-E01g18.b1D
>iib|49178 TcT-E01g19.b1D
>iib|49181 TcT-E01g21.b1D
>iib|49183 TcT-E01g22.b1D
>iib|49186 TcT-E01g23.b1D
>iib|49170 TcT-E01g5.b1D
>iib|49173 TcT-E01g6.b1D
>iib|49554 TcT-E01g7.b2D
>iib|49555 TcT-E01h1.b2D
>iib|49255 TcT-E01h11.b1D
>iib|49259 TcT-E01h13.b1d
>iib|49262 TcT-E01h14.b1d
>iib|49265 TcT-E01h15.b1d
>iib|49268 TcT-E01h16.b1d
>iib|49272 TcT-E01h17.b1d
>iib|49275 TcT-E01h18.b1d
>iib|49278 TcT-E01h19.b1d
>iib|49256 TcT-E01h2.b1d
>iib|49260 TcT-E01h20.b1d
>iib|49263 TcT-E01h21.b1d
>iib|49266 TcT-E01h22.b1d
>iib|49269 TcT-E01h23.b1d
>iib|49273 TcT-E01h24.b1d
>iib|49276 TcT-E01h3.b1d
>iib|49279 TcT-E01h4.b1d
>iib|49257 TcT-E01h5.b1d
>iib|49261 TcT-E01h6.b1d
>iib|49270 TcT-E01h9.b1d
>iib|49184 TcT-E01i10.b1D
>iib|49187 TcT-E01i11.b1D
>iib|49171 TcT-E01i13.b1D
>iib|49174 TcT-E01i14.b1D
>iib|49179 TcT-E01i16.b1D
>iib|49182 TcT-E01i17.b1D
>iib|49185 TcT-E01i18.b1D
>iib|49188 TcT-E01i19.b1D
>iib|49169 TcT-E01i20.b1D
>iib|49175 TcT-E01i22.b1D
>iib|49177 TcT-E01i24.b1D

>iib|49180 TcT-E01i3.b1D
>iib|49556 TcT-E01i4.b2D
>iib|49557 TcT-E01i6.b2D
>iib|49558 TcT-E01i7.b2D
>iib|49189 TcT-E01i8.b1D
>iib|49274 TcT-E01j1.b1d
>iib|49258 TcT-E01j13.b1d
>iib|49264 TcT-E01j15.b1d
>iib|49267 TcT-E01j16.b1d
>iib|49271 TcT-E01j17.b1d
>iib|49277 TcT-E01j19.b1d
>iib|49280 TcT-E01j2.b1d
>iib|49289 TcT-E01j22.b1d
>iib|49291 TcT-E01j23.b1d
>iib|49295 TcT-E01j3.b1d
>iib|49299 TcT-E01j4.b1d
>iib|49281 TcT-E01j5.b1d
>iib|49283 TcT-E01j6.b1d
>iib|49286 TcT-E01j7.b1d
>iib|49190 TcT-E01k10.b1D
>iib|49191 TcT-E01k12.b1D
>iib|49559 TcT-E01k14.b2D
>iib|49192 TcT-E01k16.b1D
>iib|49193 TcT-E01k18.b1D
>iib|49560 TcT-E01k19.b2D
>iib|49194 TcT-E01k20.b1D
>iib|49195 TcT-E01k21.b1D
>iib|49561 TcT-E01k22.b2D
>iib|49196 TcT-E01k23.b1D
>iib|49197 TcT-E01k5.b1D
>iib|49562 TcT-E01k9.b2D
>iib|49292 TcT-E01l1.b1d
>iib|49296 TcT-E01l11.b1d
>iib|49282 TcT-E01l13.b1d
>iib|49284 TcT-E01l14.b1d
>iib|49287 TcT-E01l15.b1d
>iib|49297 TcT-E01l19.b1d
>iib|49300 TcT-E01l2.b1d
>iib|49285 TcT-E01l21.b1d
>iib|49288 TcT-E01l24.b1d
>iib|49290 TcT-E01l3.b1d
>iib|49293 TcT-E01l4.b1d
>iib|49294 TcT-E01l5.b1d
>iib|49298 TcT-E01l6.b1d
>iib|49305 TcT-E01l8.b1D
>iib|49198 TcT-E01m10.b1D
>iib|49199 TcT-E01m12.b1D
>iib|49202 TcT-E01m14.b1D
>iib|49204 TcT-E01m15.b1D
>iib|49206 TcT-E01m16.b1D

>iib|49563 TcT-E01m2.b2D
>iib|49208 TcT-E01m21.b1D
>iib|49210 TcT-E01m23.b1D
>iib|49212 TcT-E01m4.b1D
>iib|49214 TcT-E01m5.b1D
>iib|49217 TcT-E01m6.b1D
>iib|49219 TcT-E01m7.b1D
>iib|49221 TcT-E01m8.b1D
>iib|49223 TcT-E01m9.b1D
>iib|49313 TcT-E01n12.b1D
>iib|49316 TcT-E01n13.b1D
>iib|49302 TcT-E01n16.b1D
>iib|49306 TcT-E01n17.b1D
>iib|49310 TcT-E01n19.b1D
>iib|49314 TcT-E01n2.b1D
>iib|49317 TcT-E01n20.b1D
>iib|49320 TcT-E01n22.b1D
>iib|49301 TcT-E01n24.b1D
>iib|49303 TcT-E01n3.b1D
>iib|49307 TcT-E01n6.b1D
>iib|49311 TcT-E01n8.b1D
>iib|49225 TcT-E01o10.b1D
>iib|49227 TcT-E01o11.b1D
>iib|49229 TcT-E01o12.b1D
>iib|49232 TcT-E01o13.b1D
>iib|49233 TcT-E01o14.b1D
>iib|49564 TcT-E01o19.b2D
>iib|49235 TcT-E01o2.b1D
>iib|49237 TcT-E01o20.b1D
>iib|49239 TcT-E01o21.b1D
>iib|49241 TcT-E01o22.b1D
>iib|49243 TcT-E01o24.b1D
>iib|49245 TcT-E01o3.b1D
>iib|49247 TcT-E01o4.b1D
>iib|49249 TcT-E01o6.b1D
>iib|49251 TcT-E01o7.b1D
>iib|49253 TcT-E01o8.b1D
>iib|49318 TcT-E01p11.b1D
>iib|49321 TcT-E01p12.b1D
>iib|49506 TcT-E01p13.b2D
>iib|49304 TcT-E01p14.b1D
>iib|49308 TcT-E01p16.b1D
>iib|49309 TcT-E01p17.b1D
>iib|49312 TcT-E01p18.b1D
>iib|49315 TcT-E01p19.b1D
>iib|49319 TcT-E01p2.b1D
>iib|49327 TcT-E01p23.b1D
>iib|49330 TcT-E01p24.b1D
>iib|49336 TcT-E01p6.b1D
>iib|49340 TcT-E01p8.b1D

>iib|49322 TcT-E01p9.b1D
>iib|49328 TcT-E02a10.b1D
>iib|49331 TcT-E02a11.b1D
>iib|49334 TcT-E02a12.b1D
>iib|49338 TcT-E02a16.b1D
>iib|49341 TcT-E02a17.b1D
>iib|49323 TcT-E02a18.b1D
>iib|49325 TcT-E02a2.b1D
>iib|49329 TcT-E02a22.b1D
>iib|49332 TcT-E02a3.b1D
>iib|49335 TcT-E02a4.b1D
>iib|49337 TcT-E02a6.b1D
>iib|49339 TcT-E02a7.b1D
>iib|49342 TcT-E02a9.b1D
>iib|49583 TcT-E02b2.b1D
>iib|49582 TcT-E02b3.b1D
>iib|49585 TcT-E02b6.b1D
>iib|49587 TcT-E02b7.b1D
>iib|49595 TcT-E02b8.b1D
>iib|49598 TcT-E02b9.b1D
>iib|49324 TcT-E02c10.b1D
>iib|49326 TcT-E02c11.b1D
>iib|49333 TcT-E02c14.b1D
>iib|49345 TcT-E02c19.b1D
>iib|49348 TcT-E02c2.b1D
>iib|49351 TcT-E02c21.b1D
>iib|49356 TcT-E02c3.b1D
>iib|49360 TcT-E02c4.b1D
>iib|49364 TcT-E02c5.b1D
>iib|49346 TcT-E02c7.b1D
>iib|49349 TcT-E02c8.b1D
>iib|49589 TcT-E02d1.b1D
>iib|49507 TcT-E02d10.b1D
>iib|49565 TcT-E02d11.b1D
>iib|49593 TcT-E02d2.b1D
>iib|49591 TcT-E02d4.b1D
>iib|49600 TcT-E02d5.b1D
>iib|49601 TcT-E02d6.b1D
>iib|49602 TcT-E02d8.b1D
>iib|49353 TcT-E02e11.b1D
>iib|49357 TcT-E02e12.b1D
>iib|49361 TcT-E02e13.b1D
>iib|49365 TcT-E02e15.b1D
>iib|49343 TcT-E02e16.b1D
>iib|49352 TcT-E02e2.b1D
>iib|49354 TcT-E02e21.b1D
>iib|49358 TcT-E02e22.b1D
>iib|49362 TcT-E02e23.b1D
>iib|49366 TcT-E02e4.b1D
>iib|49344 TcT-E02e5.b1D

>iib|49347 TcT-E02e6.b1D
>iib|49350 TcT-E02e7.b1D
>iib|49502 TcT-E02e8.b2D
>iib|49566 TcT-E02f10.b1D
>iib|49567 TcT-E02f11.b1D
>iib|49599 TcT-E02f2.b1D
>iib|49594 TcT-E02f3.b1D
>iib|49597 TcT-E02f4.b1D
>iib|49603 TcT-E02f5.b1D
>iib|49605 TcT-E02f7.b1D
>iib|49606 TcT-E02f8.b1D
>iib|49355 TcT-E02g1.b1D
>iib|49359 TcT-E02g10.b1D
>iib|49363 TcT-E02g11.b1D
>iib|49501 TcT-E02g13.b2D
>iib|49372 TcT-E02g15.b1D
>iib|49373 TcT-E02g16.b1D
>iib|49377 TcT-E02g18.b1D
>iib|49381 TcT-E02g19.b1D
>iib|49384 TcT-E02g2.b1D
>iib|49388 TcT-E02g20.b1D
>iib|49367 TcT-E02g22.b1D
>iib|49370 TcT-E02g23.b1D
>iib|49496 TcT-E02g24.b2D
>iib|49374 TcT-E02g3.b1D
>iib|49378 TcT-E02g4.b1D
>iib|49382 TcT-E02g5.b1D
>iib|49385 TcT-E02g6.b1D
>iib|49389 TcT-E02g7.b1D
>iib|49368 TcT-E02g8.b1D
>iib|49371 TcT-E02g9.b1D
>iib|49581 TcT-E02h1.b1D
>iib|49568 TcT-E02h11.b1D
>iib|49584 TcT-E02h2.b1D
>iib|49607 TcT-E02h7.b1D
>iib|49494 TcT-E02i1.b2D
>iib|49375 TcT-E02i10.b1D
>iib|49379 TcT-E02i11.b1D
>iib|49383 TcT-E02i12.b1D
>iib|49386 TcT-E02i13.b1D
>iib|49390 TcT-E02i14.b1D
>iib|49369 TcT-E02i15.b1D
>iib|49499 TcT-E02i16.b2D
>iib|49492 TcT-E02i17.b2D
>iib|49376 TcT-E02i18.b1D
>iib|49380 TcT-E02i19.b1D
>iib|49504 TcT-E02i2.b2D
>iib|49387 TcT-E02i20.b1D
>iib|49508 TcT-E02i22.b1D
>iib|49509 TcT-E02i23.b1D

>iib|49510 TcT-E02i24.b1D
>iib|49498 TcT-E02i3.b2D
>iib|49495 TcT-E02i4.b2D
>iib|49511 TcT-E02i5.b1D
>iib|49569 TcT-E02i8.b2D
>iib|49570 TcT-E02i9.b2D
>iib|49571 TcT-E02j10.b1D
>iib|49572 TcT-E02j12.b1D
>iib|49588 TcT-E02j2.b1D
>iib|49586 TcT-E02j4.b1D
>iib|49604 TcT-E02j8.b1D
>iib|49500 TcT-E02k10.b2D
>iib|49512 TcT-E02k11.b1D
>iib|49513 TcT-E02k17.b1D
>iib|49505 TcT-E02k18.b2D
>iib|49514 TcT-E02k19.b1D
>iib|49515 TcT-E02k20.b1D
>iib|49516 TcT-E02k21.b1D
>iib|49503 TcT-E02k23.b2D
>iib|49517 TcT-E02k6.b1D
>iib|49518 TcT-E02k7.b1D
>iib|49573 TcT-E02k7.b2D
>iib|49519 TcT-E02k8.b1D
>iib|49520 TcT-E02k9.b1D
>iib|49590 TcT-E02l1.b1D
>iib|49574 TcT-E02l11.b1D
>iib|49575 TcT-E02l12.b1D
>iib|49592 TcT-E02l4.b1D
>iib|49608 TcT-E02l5.b1D
>iib|49609 TcT-E02l7.b1D
>iib|49596 TcT-E02l8.b1D
>iib|49576 TcT-E02l9.b1D
>iib|49521 TcT-E02m1.b1D
>iib|49522 TcT-E02m10.b1D
>iib|49523 TcT-E02m11.b1D
>iib|49524 TcT-E02m12.b1D
>iib|49525 TcT-E02m14.b1D
>iib|49526 TcT-E02m18.b1D
>iib|49527 TcT-E02m19.b1D
>iib|49528 TcT-E02m20.b1D
>iib|49529 TcT-E02m21.b1D
>iib|49530 TcT-E02m23.b1D
>iib|49531 TcT-E02m4.b1D
>iib|49532 TcT-E02m7.b1D
>iib|49610 TcT-E02n1.b1D
>iib|49577 TcT-E02n10.b1D
>iib|49578 TcT-E02n12.b1D
>iib|49611 TcT-E02n2.b1D
>iib|49612 TcT-E02n3.b1D
>iib|49613 TcT-E02n4.b1D

>iib|49614 TcT-E02n5.b1D
>iib|49615 TcT-E02n6.b1D
>iib|49616 TcT-E02n7.b1D
>iib|49617 TcT-E02n8.b1D
>iib|49533 TcT-E02o1.b1D
>iib|49534 TcT-E02o10.b1D
>iib|49535 TcT-E02o13.b1D
>iib|49536 TcT-E02o14.b1D
>iib|49493 TcT-E02o17.b2D
>iib|49537 TcT-E02o18.b1D
>iib|49538 TcT-E02o19.b1D
>iib|49539 TcT-E02o2.b1D
>iib|49540 TcT-E02o21.b1D
>iib|49497 TcT-E02o23.b2D
>iib|49618 TcT-E02p1.b1D
>iib|49579 TcT-E02p11.b1D
>iib|49619 TcT-E02p5.b1D
>iib|49620 TcT-E02p6.b1D
>iib|49621 TcT-E02p7.b1D
>iib|49622 TcT-E02p8.b1D
>iib|49580 TcT-E02p9.b1D
